# The relationship between retinal layers and brain areas in asymptomatic first-degree relatives of sporadic forms of Alzheimer’s disease: an exploratory analysis

**DOI:** 10.1186/s13195-022-01008-5

**Published:** 2022-06-04

**Authors:** Inés López-Cuenca, Alberto Marcos-Dolado, Miguel Yus-Fuertes, Elena Salobrar-García, Lorena Elvira-Hurtado, José A. Fernández-Albarral, Juan J. Salazar, Ana I. Ramírez, Lidia Sánchez-Puebla, Manuel Enrique Fuentes-Ferrer, Ana Barabash, Federico Ramírez-Toraño, Lidia Gil-Martínez, Juan Arrazola-García, Pedro Gil, Rosa de Hoz, José M. Ramírez

**Affiliations:** 1 Ramon Castroviejo Institute of Ophthalmologic Research, Complutense University of Madrid, Madrid, 28040 Spain; 2 Health Research Institute of the Hospital Clínico San Carlos (IdISSC), Madrid, 28040 Spain; 3 Department of Medicine, School of Medicine, Complutense University of Madrid, Madrid, 28040 Spain; 4 Department of Neurology, Hospital Clínico San Carlos, Madrid, 28040 Spain; 5 Department of Diagnostic Imaging, Hospital Clínico San Carlos, Madrid, 28040 Spain; 6 Department of Immunology, Ophthalmology and ENT, Faculty of Optics and Optometry, Complutense University of Madrid, Madrid, 28037 Spain; 7 Preventive Medicine Service, Research Methodological Support Unit, Hospital Clínico San Carlos, Madrid, 28040 Spain; 8 Department of Endocrinology and Nutrition, Hospital Clínico San Carlos, Madrid, 28040 Spain; 9 Center for Biomedical Research Network on Diabetes and Associated Metabolic Diseases, Institute of Health Carlos III, Madrid, 28029 Spain; 10 Laboratory of Cognitive and Computational Neuroscience, Center for Biomedical Technology, Technical University of Madrid, Madrid, 28223 Spain; 11 Department of Experimental Psychology, Complutense University of Madrid, Madrid, 28040 Spain; 12 Foundation for Biomedical Research at Hospital Clínico San Carlos (FIBHCSC), Hospital Clínico San Carlos, Madrid, 28040 Spain; 13 Department of Radiology, Rehabilitation and Radiation Therapy, School of Medicine, Complutense University of Madrid, Madrid, 28040 Spain; 14 Memory Unit, Geriatrics Service, Hospital Clínico San Carlos, Madrid, 28040 Spain; 15 Department of Immunology, Ophthalmology and ENT, School of Medicine, Complutense University of Madrid, Madrid, 28040 Spain

**Keywords:** Alzheimer’s disease, Family history, ApoE ɛ4, Retina, OCT, MRI, Brain

## Abstract

**Background:**

Two main genetic risks for sporadic Alzheimer’s disease (AD) are a family history and ɛ4 allele of apolipoprotein E. The brain and retina are part of the central nervous system and share pathophysiological mechanisms in AD.

**Methods:**

We performed a cross-sectional study with 30 participants without a family history of sporadic AD (FH−) and noncarriers of ApoE ɛ4 (ApoE ɛ4−) as a control group and 34 participants with a family history of sporadic AD (FH+) and carriers of at least one ɛ4 allele (ApoE ɛ4+). We analyzed the correlations between macular volumes of retinal layers and thickness of the peripapillary retinal nerve fiber layer (pRNFL) measured by optical coherence tomography (OCT) with the brain area parameters measured by magnetic resonance imaging (MRI) in participants at high genetic risk of developing AD (FH+ ApoE ɛ4+).

**Results:**

We observed a significant volume reduction in the FH+ ApoE ɛ4+ group compared with the control group in some macular areas of (i) macular RNFL (mRNFL), (ii) inner plexiform layer (IPL), (iii) inner nuclear layer (INL), and (iv) outer plexiform layer (OPL). Furthermore, in the FH+ ApoE ɛ4+ group, the retinal sectors that showed statistically significant volume decrease correlated with brain areas that are affected in the early stages of AD. In the same group, the peripapillary retinal nerve fiber layer (pRNFL) did not show statistically significant changes in thickness compared with the control group. However, correlations of these sectors with the brain areas involved in this disease were also found.

**Conclusions:**

In cognitively healthy participants at high genetic risk of developing sporadic forms of AD, there are significant correlations between retinal changes and brain areas closely related to AD such as the entorhinal cortex, the lingual gyrus, and the hippocampus.

**Supplementary Information:**

The online version contains supplementary material available at 10.1186/s13195-022-01008-5.

## Background

Alzheimer’s disease (AD) is a neurodegenerative disease and the most common cause of dementia [1]. The progressive accumulation of amyloid-beta (Aβ) protein (plaques) outside the neurons [2] and the accumulation of tau protein (tangles) within the neurons [1] are the hallmarks of this disease. Aβ aggregation starts in the anterior cingulate cortex and the precuneus [3, 4], while tau tangles are located in the entorhinal cortex, the hippocampus, and adjacent limbic structures in milder cases [5]. The use of biomarkers such as quantification of Aβ and tau levels in cerebrospinal fluid (CSF) or the increased deposition of tau tangles and accumulation of Aβ plaques revealed by positron emission tomography (PET) are biomarkers that help determine the clinical stage of AD; however, they are limited because they require invasive techniques and expensive diagnostic tools [6].

The retina and the brain are part of the central nervous system, and both have a common embryological origin [7]. Currently, it is known that there is a relationship between age-related retinal neurodegenerative diseases and brain neurodegenerative diseases, including AD [8]. In addition, in the retina, protein deposits have been detected in both AD animal models and in vivo and postmortem eyes from human AD patients [9–11], with the retina having important diagnostic implications in this disease. Through optical coherence tomography (OCT), a reliable non-invasive diagnostic tool that is commonly used in ophthalmology to visualize and analyze the retinal layers, retinal changes have been observed in different stages of AD. In a previous work using OCT, we have demonstrated that, in preclinical stages, participants with a high genetic risk of developing AD show a significant thinning in several retinal layers in the macular region [12]. In patients with mild AD, thinning also occurs principally in the macular region [13–15]; when the disease progresses to a moderate stage, these changes are reflected in the peripapillary region [16].

A direct correlation has been observed between the volumes of brain areas measured by magnetic resonance imaging (MRI) and the thickness of specific retina regions using OCT in nondemented older adults [6, 17]. In addition, both family history of the disease (FH+) and ApoE ɛ4 genotype (ApoE ɛ4+) potentiate each other, contributing to the thinning of the cortex in the hippocampal region [18]. The purpose of our study was to analyze the correlations between the macular volumes of all the retinal layers and the thickness of the peripapillary retinal nerve fiber layer (pRNFL) measured by OCT, with the volumes and thickness of different brain areas measured by MRI in participants at high genetic risk of developing AD (FH+ ApoE ɛ4+) compared with a control group (FH− ApoE ɛ4−).

## Methods

### Participants

The participants were recruited from the study “The cognitive and neurophysiological characteristics of participants at high genetic risk of developing dementia: a multidimensional approach” (COGDEM Study, PSI2015-68793-C3-2-R, Ministry of Economy and Competitiveness) (*n* = 251). In this study, participants were recruited in different local hospitals and advertisements were published in different professional associations such as the “Asociación Española de Ingenieros de Telecomunicación Delegación de Madrid.” The general inclusion criteria are listed below: (i) age between 45 and 75 years; (ii) absence of psychiatric or neurological disorders; (iii) absence of evidence of infection, infarction, or focal lesions on T2-weighted magnetic resonance imaging (MRI); (iv) absence of substance addictions such as alcohol or chronic use of anxiolytic, neuroleptic, narcotic, anticonvulsant, or sedative-hypnotic drugs; and (v) absence of memory complaints. In addition, all participants underwent a neuropsychological assessment including analysis of declarative memory with the Logical Memory (immediate and delayed) and digits (forward and backward) and the Word List of the Wechsler Memory Scale-III- and everyday memory with the Rivermead Behavioural Memory Test. Subjective cognitive impairment was assessed with the MFE and a series of questions (neuropsychological interview) on possible impairment of different cognitive functions

**Fig. 1:**
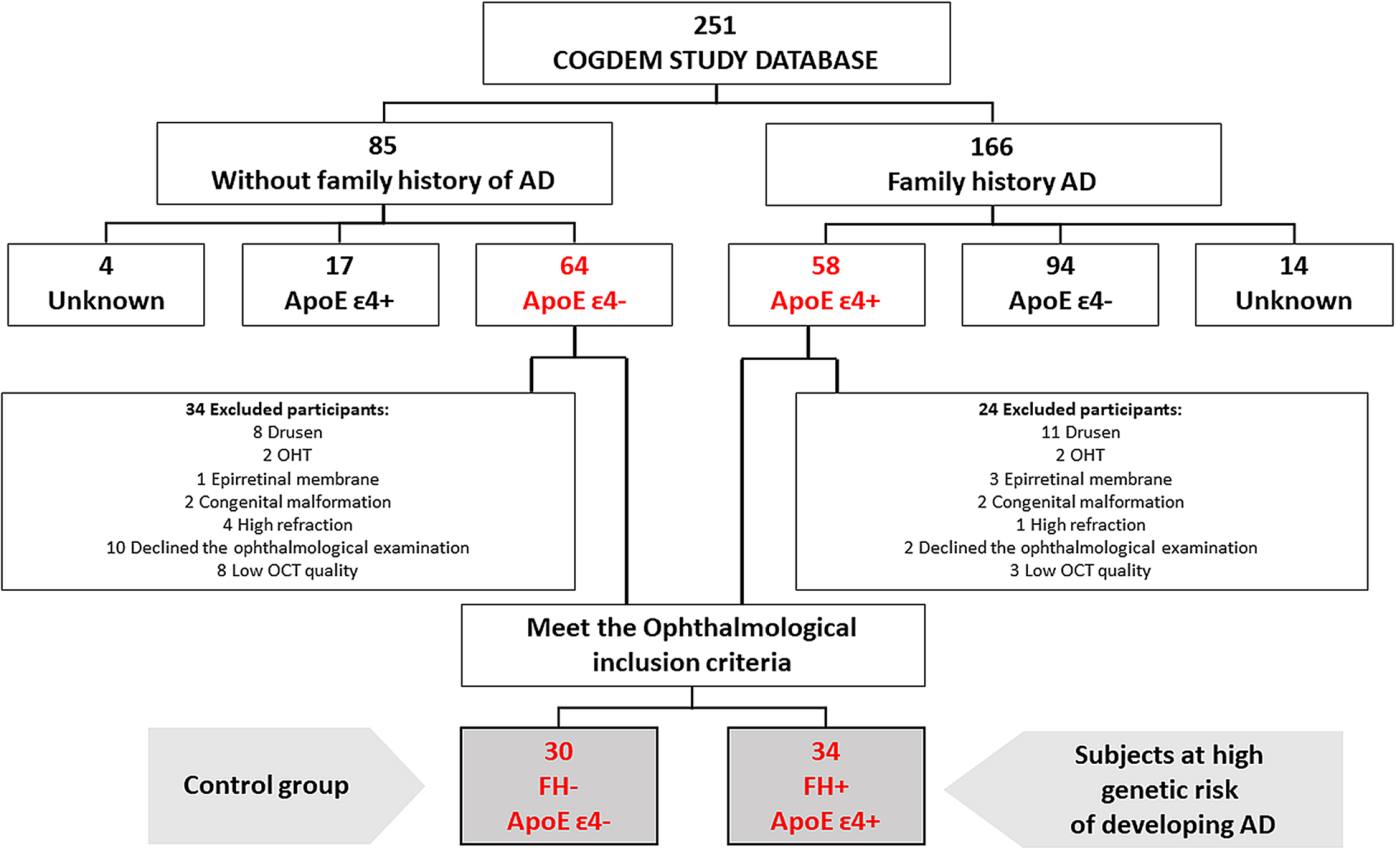
Flow diagram of subject selection. COGDEM, “The cognitive and neurophysiological characteristic of participants at high risk of developing dementia: a multidimensional approach”; FH−, participants without a family history of Alzheimer’s disease; FH+, participants with a family history of AD; ApoE ɛ4–, noncarriers of ɛ4 allele; ApoE ɛ4+, carriers of at least one ɛ4 allele. In gray are the participants who participated in the ophthalmological study and were included in this study. OHT, ocular hypertension; OCT, optical coherence tomography

We analyzed two groups. As a control group (*n* = 30), we selected from the COGDEM study database those participants with no family history of sporadic senile onset AD. These participants had the following characteristics: (i) they were cognitively healthy, (ii) they had no first-degree family history of AD (FH−), (iii) they were noncarriers of the ApoE ɛ4 allele (ApoE ɛ4−), and (iv) they had no memory complaints. The group at high genetic risk of developing AD (*n* = 34), matched with the control group in terms of age and socioeconomic status, consisted of participants with the following features: (i) cognitively healthy, (ii) first-degree family history of senile AD (FH+) (father or mother affected by the disease), (iii) carrier of at least one ɛ4 allele for the ApoE gene (ApoE ɛ4+), and (iv) no memory complaints. Figure 1 shows a flow diagram of the patient selection.

In both groups, Mini-Mental State Examination (MMSE) scores were normal (above 26) and participants had no previous history of neurological or psychiatric disorder.

The research followed the tenets of the Declaration of Helsinki and the study protocol was approved by the Local Ethics Committee (Hospital Clinic San Carlos) with the internal code 18/422-E_BS. All participants signed written informed consent to be enrolled in the study.

### Optical coherence tomography (OCT)

After an ophthalmological examination performed at the Clinic of the Ramon Castroviejo Institute of Ophthalmic Research in Madrid, which included visual acuity, slit-lamp examination, applanation tonometry (Perkins MKII tonometer, Clement Clarke International, Essex, UK), and a dilated funduscopy, one eye of each patient was randomly selected. All the participants met the following ophthalmological inclusion criteria: (i) free from ocular disease or posterior pole pathology (macular degeneration, drusen, glaucoma, or suspicion, epiretinal membrane, and congenital malformation), (ii) having a best corrected visual acuity more than 20/40, (iii) having less than ± 5 D spherocylindrical refractive error, and (iv) having an intraocular pressure less than 20 mmHg.

To analyze the volumes of the macular region, OCT imaging was performed with Spectralis OCT (Heidelberg Engineering, Heidelberg, Germany), following the OCT protocol previously published [12].

**Fig. 2:**
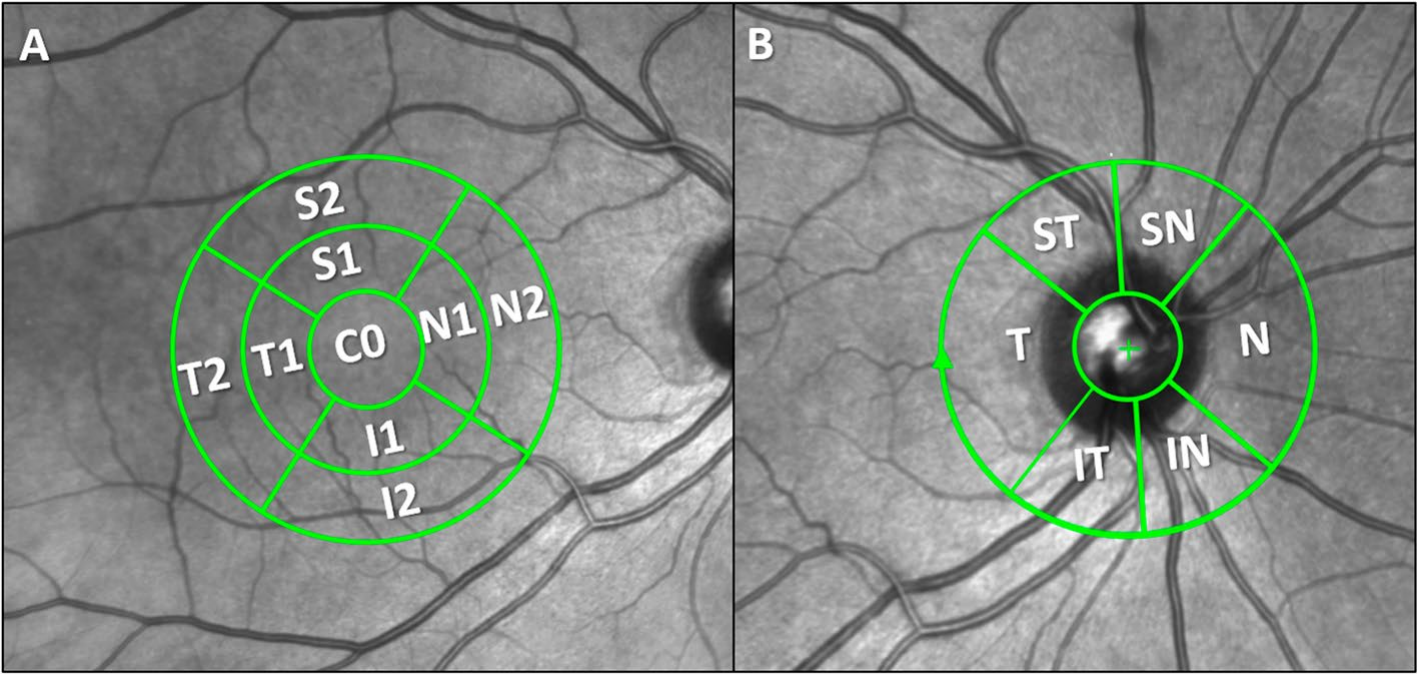
Optical coherence tomography (OCT) report of the retinal macular volume and peripapillary thickness. **A** Concentric macular rings. **B** Peripapillary sectors. C0 central macular sector, N1 nasal sector of the inner macular ring, I1 inferior sector of the inner macular ring, T1 temporal sector of the inner macular ring, S1 superior sector of the inner macular ring, N2 nasal sector of the outer macular ring, I2 inferior sector of the outer macular ring, T2 temporal sector of the outer macular ring, S2 superior sector of the outer macular ring, ST superotemporal, SN superonasal, N nasal, IN inferonasal, IT inferotemporal, T temporal

Heidelberg segmentation software (Heidelberg, Germany, version 1.10.4.0) allowed us to measure the total retinal volume and the volume of each retinal layer in the macular area. To avoid segmentation problems, all automatic segmentations were checked by the same optometrist (IL-C) and modified manually if an error was found. The macular volume of the following retinal layers was analyzed: retinal nerve fiber layer (RNFL), ganglion cell layer (GCL), inner plexiform layer (IPL), inner nuclear layer (INL), outer plexiform layer (OPL), outer nuclear layer (ONL), and retinal pigment epithelium (RPE). The macular area was analyzed according to the standard Early Treatment Diabetic Retinopathy Study (ETDRS) macular grid (a foveal area of 1 mm in diameter, 1–3 mm around the fovea in the inner ring, and 3–6 mm for the outer ring) [19] (Fig. 2A). A peripapillary RNFL (pRNFL) thickness analysis was carried out in six sectors (temporal (T), superotemporal (ST), inferotemporal (IT), nasal (N), inferonasal, and superonasal (SN) (Fig. 2B). An average of all sectors was also obtained (global). In this study, we included macular volume scans with a minimum signal-to-noise ratio of 25 and an average of 16 B-scans. The pRNFL scans have a minimum signal-to-noise ratio of 20 and an average of at least 40 B-scans. According to the calibration provided by the manufacturers, the thickness measurements were provided in microns and the volume measurements in mm^3^.

### Magnetic resonance imaging (MRI)

**Fig. 3:**
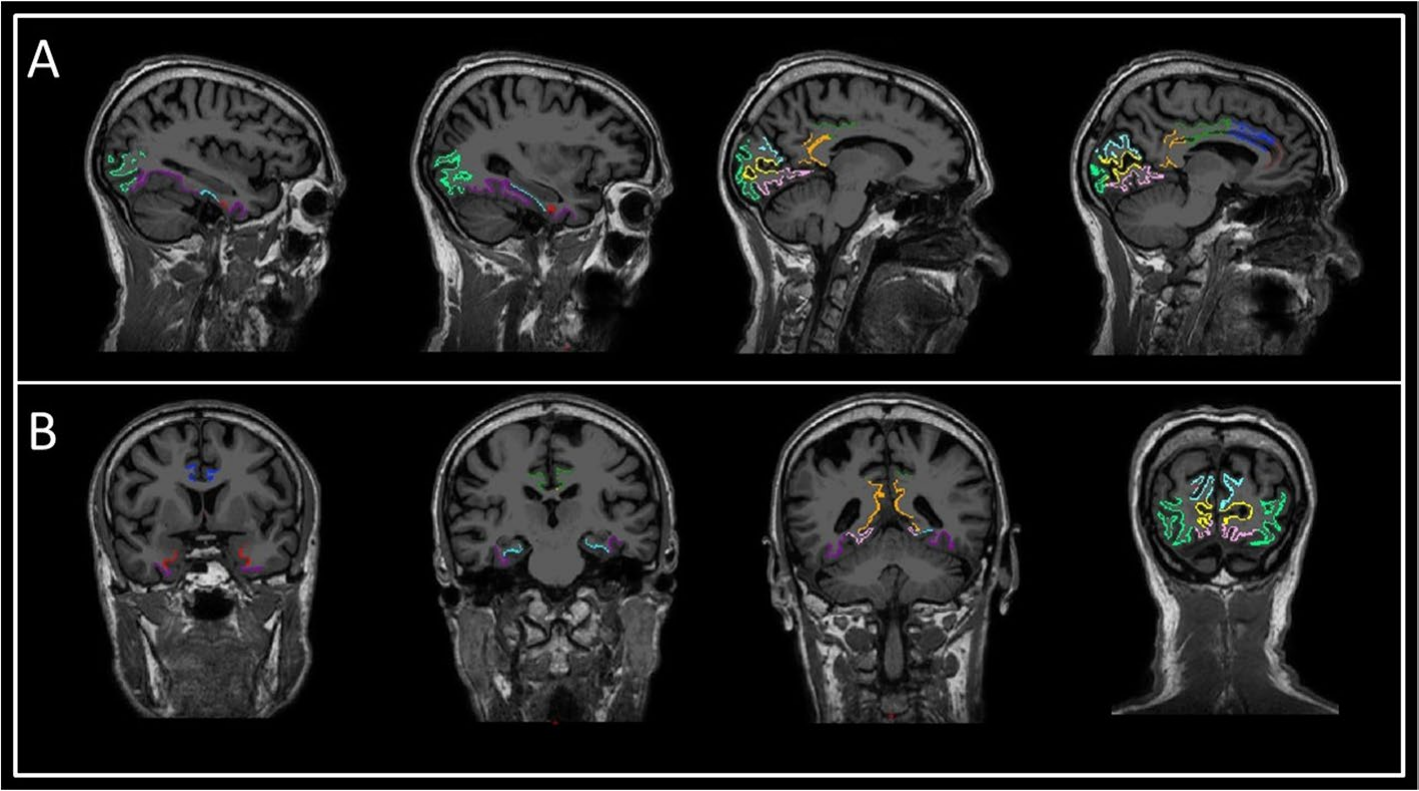
Sagittal (**A**) and axial slices (**B**) from the 3DT1 sequence of a study individual. The evaluated cortical regions are represented as a colored overlay: entorhinal (red), parahippocampal (blue), latero-occipital (light green), pericalcarine (yellow), lingual (pink), cuneus (light blue), rostral anterior cingulate (brown), caudal anterior cingulate (dark blue), posterior cingulate (green), and isthmus cingulate (orange)

For each subject, MRI were acquired using a 1.5-Tesla scanner (General Electric Medical Systems, Waukesha, WI, USA) model with HDxt release 16.0 and an eight-channel, high-resolution head coil. A high-resolution antenna was employed together with a homogenization Phased array Uniformity Enhancement filter (Fast Spoiled Gradient Echo sequence, TR/TE/TI=11.2/4.2/450 ms; flip angle 12°; 1 mm slice thickness, 256×256 matrix and FOV 25 cm). In addition to whole-head 3D fast spoiled ir-prepped gradient-echo T1-weighted 1-mm^3^ isotropic sequence, 3D CUBE FLAIR T2-weighted 1.6-mm-thickness sequence and 2D gradient-echo T2 sequence, DTI images were also obtained. T2 sequences do not contribute to the study except to rule out vascular lesions and vascular load or other differential diagnostic entities, apart from being necessary for the overall volumetric assessment (Fig. 3A, B).

Each T1-weighted MRI image was processed using FreeSurfer 6.0 *recon-all* procedure [20–24]. This procedure performs a motion correction, corrects for intensity nonuniformity, and performs an intensity normalization. Then, it performs a segmentation of the different brain tissues and it constructs a cortical surface mesh for each T1. It registers an inflated sphere version of this cortical mesh to a common surface-space and it uses the Desikan-Killiany anatomical atlas [25] to assign a neuroanatomical label to each native brain voxel. Finally, FreeSurfer 6.0 was used to obtain the volume and the cortical thickness of different areas of interest.

### Allelic characterization

APOE polymorphism (haplotype) was determined by an analysis of the genotype of the two polymorphisms (SNPs) that determine it: rs7412 and rs429358. The DNA was extracted from peripheral leukocytes using DNAzol® Genomic DNA Isolation Reagent (Molecular Research Center, Inc., Cincinnati, OH, USA), following the protocol of the manufacturer. ApoE alleles were determined using TaqMan assay technology on an Applied Biosystems 7500 Fast Real-Time PCR machine (Applied Biosystems, Foster City, CA, USA). A genotyping call rate of over 95% per plate, sample controls for each genotype, and negative sample controls were included in each assay. Three well-differentiated genotyping clusters for each SNP were required to validate the results. Intra- and interplate duplicates of several DNA samples were also included.

### Statistical analysis

Statistical analysis was carried out in SPSS 25.0 (SPSS Inc., Chicago, IL, USA). Data are reported as the median (interquartile range). The differences between study groups (FH− ApoE ɛ4− and FH− ApoE ɛ4+) in qualitative variables were analyzed using a chi-squared test. We first compared macular area volumes and peripapillary nerve fiber layer thicknesses between the study groups using the Mann–Whitney *U* test. Secondly, we compared the volumes of brain areas between the study groups also using the Mann–Whitney *U* test. Finally, partial correlation controlling for age using Pearson’s correlation coefficient was applied to study the possible association between macular and peripapillary retinal sectors and brain structures that are mainly related to AD. A colorimetric scale has been applied to the degree of correlation of the variables, where low correlations (*r* with values between 0.38 and 0.60) have a yellowish color, medium correlations (*r* with values between 0.60 and 0.72) have orange colors, and strong correlations (*r* with values between 0.72 and 1.00) have reddish tones. A *p*-value <0.05 was considered statistically significant.

## Results

### Demographic data

Demographic data for the FH− ApoE ɛ4− and FH+ ApoE ɛ4+ groups are shown in Table 1.

**Table 1:** Demographic data of participants

	FH– ApoE ɛ4–	FH+ ApoE ɛ4+	***p***-value
**Number of participants (*n*)**	30	34	
**Age (years)**	60.0 (54.0–64.5)	57.0 (54.0–61.0)	0.210
**Male/female**	12/18	11/23	**0.273**^**1**^/0.040*^,1^
**MMSE**	29.0 (29.0–29.0)	29.0 (28.0–29.0)	

Median (interquartile range); *p < 0.05. Mann–Whitney *U* and chi-square tests; ^1^*p*-value of different sex in the same group. *FH–* participants without a family history of AD, *FH+* participants with a family history of AD, *ApoE ɛ4–* noncarriers of ApoE ɛ4, *ApoE ɛ4+* carriers of ApoE ɛ4, *MMSE* Mini-Mental State Examination

There were no differences between study groups in terms of age and MMSE (*p* > 0.05). The FH− ApoE ɛ4− group showed a median age of 60.0 (54.0–64.5) years and the FH+ ApoE ɛ4+ group had a median age of 57.0 (54.0–61.0) years. The median MMSE scores were 29.0 (29.0–29.0) in the FH− ApoE ɛ4− group and 29.0 (28.0–29.0) in the FH+ ApoE ɛ4+ group.

In terms of sex, there were statistically significant differences (*p* < 0.05) between the numbers of males (11) and females (23) participating in the ApoE ɛ4+ group.

### Magnetic resonance imaging

Regarding the cortical thickness, there were no significant differences in any of the regions analyzed between the study groups (*p* > 0.05) (Table 2).

**Table 2:** Thickness of the different cortical regions between study groups

Cortical regions	FH− ApoE ε4−	FH+ ApoE ε4+	***p***-value
**Lingual right**	1.980 (0.103)	1.985 (0.115)	0.873
**Lingual left**	1.965 (0.101)	1.950 (0.094)	0.549
**Rostral anterior** **cingulate right**	2.827 (0.208)	2.861 (0.309)	0.635
**Rostral anterior** **cingulate left**	2.704 (0.205)	2.721 (0.198)	0.764
**Caudal anterior** **cingulate right**	2.466 (0.283)	2.440 (0.228)	0.708
**Caudal anterior** **cingulate left**	2.527 (0.231)	2.526 (0.274)	0.995
**Posterior cingulate right**	2.343 (0.241)	2.358 (0.122)	0.760
**Posterior cingulate left**	2.329 (0.118)	2.298 (0.169)	0.444
**Isthmus cingulate right**	2.301 (0.180)	2.398 (0.195)	0.059
**Isthmus cingulate left**	2.350 (0.226)	2.312 (0.188)	0.502
**Entorhinal right**	3.558 (0.336)	3.518 (0.263)	0.621
**Entorhinal left**	3.452 (0.384)	3.443 (0.346)	0.933
**Fusiform right**	2.619 (0.102)	2.641 (0.114)	0.467
**Fusiform left**	2.605 (0.101)	2.628 (0.0988)	0.390
**Global cortical right**	2.360 (0.074)	2.363 (0.068)	0.860
**Global cortical left**	2.368 (0.076)	2.357 (0.075)	0.602

Data are expressed as mean and typical deviation. *p < 0.05. Mann–Whitney *U* test. *FH–* participants without a family history of AD, *FH+* participants with a family history of AD, *ApoE ɛ4–* noncarriers of ApoE ɛ4, *ApoE ɛ4+* carriers of ApoE ɛ4. Measurements are expressed in millimeters

Regarding the volume of the different brain regions studied, there were no significant differences in any of the regions analyzed between the study groups (*p* > 0.05) (Table 3).

**Table 3:** Volume of the different brain regions between study groups

Brain regions	FH– ApoE ε4–	FH+ ApoE ε4+	***p***-value
**Parahippocampal right**	1825.89 (211.59)	1902.62 (217.98)	0.187
**Parahippocampal left**	2053.15 (293.95)	2086.86 (293.95)	0.634
**Entorhinal right**	2048.11 (286.42)	2120.97 (352.46)	0.402
**Entorhinal left**	2181.70 (332.07)	2165.55 (308.12)	0.851
**Lingual gyrus right**	6357.89 (880.20)	6417.83 (1047.83)	0.872
**Lingual gyrus left**	6004.44 (987.93)	6023.17 (835.08)	0.929
**Pericalcarine right**	2412.85 (497.76)	2396.17 (466.96)	0.898
**Pericalcarine left**	2119.63 (452.79)	2142.48 (395.24)	0.841
**Lateral occipital right**	11,388.93 (1302.19)	11,381.93 (1506.77)	0.985
**Lateral occipital left**	11,218.59 (1247.33)	11,108.52 (1237.37)	0.742
**Cuneus right**	3032.56 (442.16)	3095.03 (574.73)	0.652
**Cuneus left**	2811.04 (450.16)	2874.31 (517.92)	0.629
**Medial temporal right**	8014.46 (631.63)	8127.23 (761.61)	0.551
**Medial temporal left**	8158.11 (729.41)	8191.88 (791.15)	0.869
**Occipital right**	23,210.22 (2589.45)	23,290.97 (2853.82)	0.912
**Occipital left**	22,153.70 (2482.72)	22,151.48 (2541.49)	0.997
**Amygdala right**	1502.15 (247.41)	1467.93 (215.52)	0.583
**Amygdala left**	1298.38 (186.45)	1292.43 (147.13)	0.895
**Ventral diencephalon right**	3715.45 (349.01)	3701.44 (341.39)	0.880
**Ventral diencephalon left**	3711.05 (367.22)	3757.41 (386.31)	0.648
**Hippocampus right**	4140.46 (367.54)	4103.64 (431.50)	0.733
**Hippocampus left**	3923.25 (346.95)	3939.47 (413.06)	0.875
**Intracranial global**	1,438,163.2 (162,347.9)	1,415,555.1 (119,222.5)	0.553

Data are expressed as mean and typical deviation. *p < 0.05. Mann–Whitney *U* test. *FH–* participants without a family history of AD, *FH+* participants with a family history of AD, *ApoE ɛ4–* noncarriers of ApoE ɛ4, *ApoE ɛ4+* carriers of ApoE ɛ4. Measurements are expressed in cubic millimeter

### Retinal layer volume between groups

Regarding the total retinal volume, there were no significant differences in any of the sectors analyzed between the study groups (*p* > 0.05).

In the macular retinal nerve fiber layer (mRNFL), the foveal area (C0) showed a significant reduction in volume (*p* < 0.05) in the FH+ ApoE ɛ4+ group (0.009 (0.008–0.010)) mm^3^, compared with the FH– ApoE ɛ4– group (0.010 (0.009–0.011)) mm^3^ (Table 4).

**Table 4:** Volumes of retinal sectors of each retinal layer

Retinal layer	Ring	Sector	FH– ApoE ε4–	FH+ ApoE ε4+	***p***-value
**Total retina**		C0	0.220 (0.208–0.232)	0.218 (0.207–0.222)	0.213
	**Inner**	N1	0.545 (0.537–0.562)	0.543 (0.533–0.558)	0.522
		S1	0.540 (0.527–0.555)	0.541 (0.532–0.553)	0.701
		T1	0.518 (0.503–0.529)	0.517 (0.507–0.529)	0.788
		I1	0.538 (0.523–0.551)	0.533 (0.523–0.549)	0.657
	**Outer**	N2	1.681 (1.618–1.745)	1.678 (1.628–1.734)	0.898
		S2	1.564 (1.520–1.645)	1.582 (1.559–1.605)	0,545
		T2	1.490 (1.439–1.571)	1.495 (1.467–1.539)	0.904
		I2	1.532 (1.488–1.596)	1.529 (1.495–1.575)	0.752
**mRNFL**		C0	0.010 (0.009–0.011)	0.009 (0.008–0.010)	0.044*
	**Inner**	N1	0.032 (0.031–0.035)	0.033 (0.031–0.035)	0.817
		S1	0.038 (0.036–0.042)	0.039 (0.036–0.041)	0.586
		T1	0.027 (0.027–0.028)	0.027 (0.026–0.028)	0.939
		I1	0.038 (0.035–0.041)	0.041 (0.038–0.042)	0.104
	**Outer**	N2	0.249 (0.233–0.274)	0.270 (0.236–0.286)	0.205
		S2	0.196 (0.184–0.217)	0.196 (0.186–0.217)	0.622
		T2	0.101 (0.095–0.111)	0.101 (0.095–0.106)	0.264
		I2	0.207 (0.191–0.240)	0.217 (0.196–0.233)	0.513
**GCL**		C0	0.012 (0.010–0.015)	0.011 (0.009–0.013)	0.112
	**Inner**	N1	0.083 (0.078–0.088)	0.082 (0.074–0.085)	0.321
		S1	0.085 (0.079–0.088)	0.082 (0.077–0.086)	0.247
		T1	0.074 (0.072–0.079)	0.075 (0.068–0.077)	0.355
		I1	0.082 (0.080–0.085)	0.082 (0.077–0.085)	0.478
	**Outer**	N2	0.204 (0.190–0.223)	0.207 (0.186–0.212)	0.369
		S2	0.186 (0.170–0.196)	0.180 (0.164–0.196)	0.548
		T2	0.186 (0.170–0.207)	0.180 (0.170–0.196)	0.504
		I2	0.180 (0.172–0.191)	0.175 (0.159–0.186)	0.087
**IPL**		C0	0.016 (0.015–0.019)	0.015 (0.014–0.017)	0.077
	**Inner**	N1	0.069 (0.066–0.071)	0.066 (0.063–0.070)	0.026*
		S1	0.068 (0.064–0.069)	0.064 (0.062–0.068)	0.068
		T1	0.066 (0.061–0.068)	0.063 (0.061–0.068)	0.170
		I1	0.066 (0.064–0.069)	0.064 (0.061–0.066)	0.019*
	**Outer**	N2	0.170 (0.148–0.175)	0.159 (0.143–0.166)	0.044*
		S2	0.154 (0.143–0.164)	0.148 (0.138–0.160)	0.142
		T2	0.178 (0.163–0.191)	0.170 (0.159–0.180)	0.103
		I2	0.154 (0.146–0.159)	0.143 (0.131–0.154)	0.006*
**INL**		C0	0.016 (0.014–0.020)	0.014 (0.013–0.017)	0.041*
	**Inner**	N1	0.068 (0.061–0.072)	0.065 (0.061–0.068)	0.313
		S1	0.063 (0.061–0.066)	0.065 (0.061–0.068)	0.449
		T1	0.060 (0.056–0.064)	0.060 (0.057–0.063)	0.925
		I1	0.067 (0.061–0.071)	0.065 (0.063–0.071)	0.792
	**Outer**	N2	0.186 (0.175–0.192)	0.180 (0.170–0.191)	0.177
		S2	0.167 (0.158–0.175)	0.164 (0.159–0.175)	0.929
		T2	0.175 (0.164–0.186)	0.170 (0.164–0.180)	0.463
		I2	0.170 (0.159–0.180)	0.164 (0.154–0.170)	0.045*
**OPL**		C0	0.020 (0.019–0.022)	0.020 (0.018–0.024)	0.710
	**Inner**	N1	0.050 (0.047–0.077)	0.052 (0.047–0.061)	0.463
		S1	0.049 (0.045–0.060)	0.049 (0.044–0.062)	0.984
		T1	0.048 (0.045–0.052)	0.049 (0.045–0.052)	0.441
		I1	0.053 (0.049–0.081)	0.049 (0.046–0.073)	0.089
	**Outer**	N2	0.162 (0.147–0.191)	0.159 (0.143–0.164)	0.203
		S2	0.138 (0.131–0.154)	0.140 (0.133–0.154)	0.476
		T2	0.148 (0.138–0.159)	0.143 (0.138–0.154)	0.554
		I2	0.159 (0.143–0.171)	0.143 (0.138–0.155)	0.007*
**ONL**		C0	0.075 (0.071–0.080)	0.077 (0.071–0.080)	0.861
	**Inner**	N1	0.115 (0.090–0.123)	0.119 (0.105–0.126)	0.236
		S1	0.108 (0.102–0.118)	0.112 (0.099–0.120)	0.909
		T1	0.115 (0.108–0.123)	0.118 (0.108–0.121)	0.736
		I1	0.104 (0.072–0.112)	0.106 (0.088–0.115)	0.367
	**Outer**	N2	0.281 (0.257–0.307)	0.302 (0.265–0.325)	0.110
		S2	0.307 (0.289–0.333)	0.323 (0.292–0.337)	0.198
		T2	0.292 (0.270–0.307)	0.305 (0.286–0.329)	0.062
		I2	0.260 (0.231–0.281)	0.270 (0.254–0.304)	0.070
**RPE**		C0	0.013 (0.012–0.013)	0.013 (0.012–0.013)	0.211
	**Inner**	N1	0.024 (0.022–0.025)	0.024 (0.022–0.027)	0.164
		S1	0.024 (0.022–0.025)	0.024 (0.022–0.025)	0.498
		T1	0.022 (0.022–0.024)	0.022 (0.022–0.025)	0.505
		I1	0.022 (0.022–0.024)	0.023 (0.022–0.025)	0.175
	**Outer**	N2	0.069 (0.064–0.074)	0.069 (0.064–0.074)	0.480
		S2	0.069 (0.069–0.074)	0.072 (0.069–0.074)	0.690
		T2	0.069 (0.064–0.069)	0.069 (0.064–0.069)	0.648
		I2	0.069 (0.064–0.069)	0.069 (0.062–0.070)	0.859

Median (interquartile range); *p < 0.05, in bold. Mann–Whitney *U* test. *FH*− participants without a family history of AD, *FH+* participants with a family history of AD, *ApoE ɛ4*− noncarriers of ApoE ɛ4, *ApoE ɛ4+* carriers of ApoE ɛ4, *mRNFL* macular retinal nerve fiber layer, *GCL* ganglion cell layer, *IPL* inner plexiform layer, *INL* inner nuclear layer, *OPL* outer plexiform layer, *ONL* outer nuclear layer, *RPE* retinal pigment epithelium, *C0* central macular sector, *N1* nasal sector of the inner macular ring, *I1* inferior sector of the inner macular ring, *T1* temporal sector of the inner macular ring, *S1* superior sector of the inner macular ring, *N2* nasal sector of the outer macular ring, *I2* inferior sector of the outer macular ring, *T2* temporal sector of the outer macular ring, *S2* superior sector of the outer macular ring. Measurements are expressed in cubic millimeter

In the GCL, the FH+ ApoE ɛ4+ group showed a slight, nonsignificant volume reduction of all sectors in comparison to the FH– ApoE ɛ4– (Table 4).

In the IPL, the FH+ ApoE ɛ4+ group showed a statistically significant volume reduction (*p* < 0.05) in the inferior sectors, both in the inner macular ring (I1) (0.064 (0.061–0.066)) mm^3^ and the outer macular ring (I2) (0.143 (0.131–0.154)) mm^3^, with respect to the FH– ApoE ɛ4– group (0.066 (0.064–0.069)) mm^3^ and (0.154 (0.146–0.159)) mm^3^. In the nasal sectors, we also observed this volume reduction (*p* < 0.05) in the inner (N1) (0.066 (0.063–0.070)) mm^3^ and outer (N2) (0.159 (0.143–0.166)) mm^3^ macular rings in the FH+ ApoE ɛ4+ group in comparison to the FH– ApoE ɛ4– group (0.069 (0.066–0.071)) and (0.170 (0.148–0.175)) mm^3^ (Table 4).

In the INL, the FH+ ApoE ɛ4+ group, in comparison to FH– ApoE ɛ4–, showed a significant volume decrease (*p* < 0.05) in the foveal sector (C0) (0.014 (0.013–0.017)) mm^3^ vs. (0.016 (0.014–0.020)) mm^3^ and in the outer macular ring in the inferior sector (I2) (0.164 (0.154–0.170)) mm^3^ vs. (0.170 (0.159–0.180)) mm^3^ (Table 4).

In the OPL, the FH+ ApoE ɛ4+ group showed a significant volume reduction (*p* < 0.05) in the outer macular ring in the inferior sector (I2) (0.143 (0.138–0.155)) mm^3^ compared with the FH– ApoE ɛ4– group (0.159 (0.143–0.171)) mm^3^ (Table 4).

In ONL, EPR, and pRNFL, there were no statistically significant differences (*p* > 0.05) in any of the sectors analyzed between the study groups (Table 4).

### Correlations between the retina and brain in the studied groups

We studied Pearson correlations between the retina (macular sectors and pRNFL) and specific brain structures in each study.

All significant Pearson correlations can be found in Tables 4, 5, and 6 and S1–S8 and Figs. 4, 5, 6 and 7 and supplementary figs. 1, 2, 3 and 4.

**Table 5:**
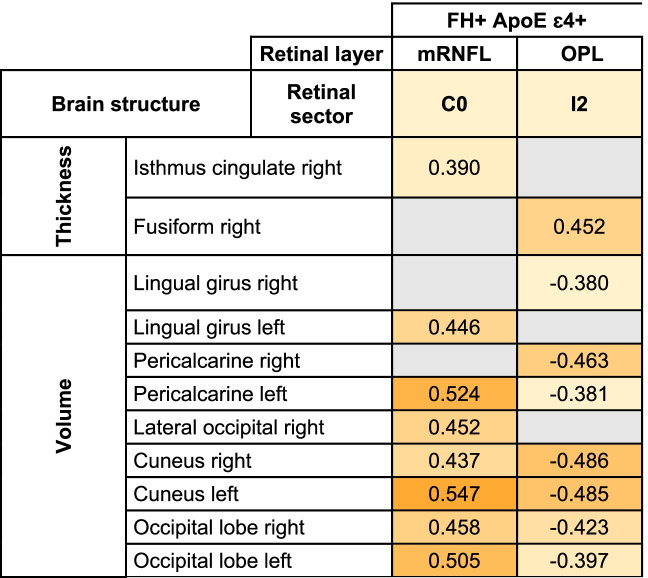
Significant age-adjusted correlations between retinal sector volumes and the volumes and thickness of brain structures in participants with high genetic risk of developing AD. A colorimetric scale has been applied to the degree of correlation of the variables

*FH*+: participants with a family history of AD, *RNFL* retinal nerve fiber layer; *OPL* Outer plexiform layer; right: right hemisphere and left: left hemisphere

**Table 6:**
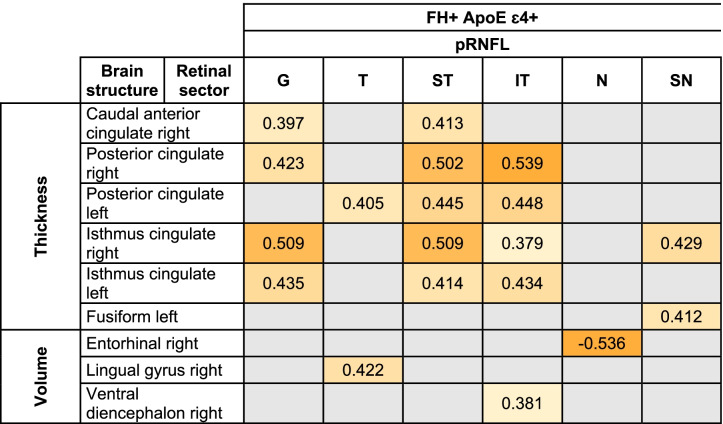
Significant age-adjusted correlations between pRNFL thickness in different sectors and thickness and volume of brain structures in participants with a high risk of developing AD. A colorimetric scale has been applied to the degree of correlation of the variables

*FH*+ participants with a family history of AD, *ApoE ɛ4+* carriers of ApoE ɛ4, *pRNFL* peripapillary retinal nerve fiber layer, *G* global, *T* temporal, *ST* superotemporal, *IT* inferotemporal, *N* nasal, *SN* superonasal, *right* right hemisphere, *left* left hemisphere

**Fig. 4:**
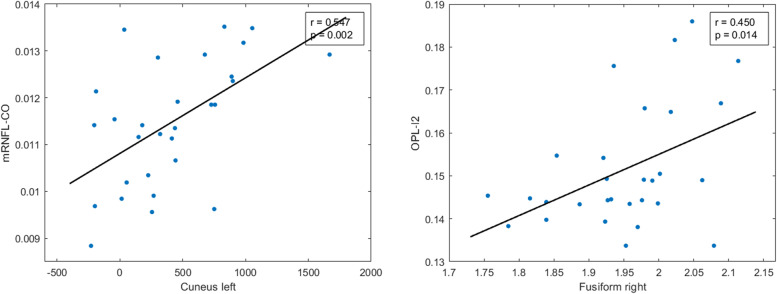
Scatter plots of some statistically significant age-adjusted correlations between retinal sector volumes and volumes and thickness of brain structures in participants with high genetic risk of developing AD. mRNFL macular retinal nerve fiber layer, C0 central macular sector, OPL outer plexiform layer, I2 inferior sector of the outer macular ring

**Fig. 5:**
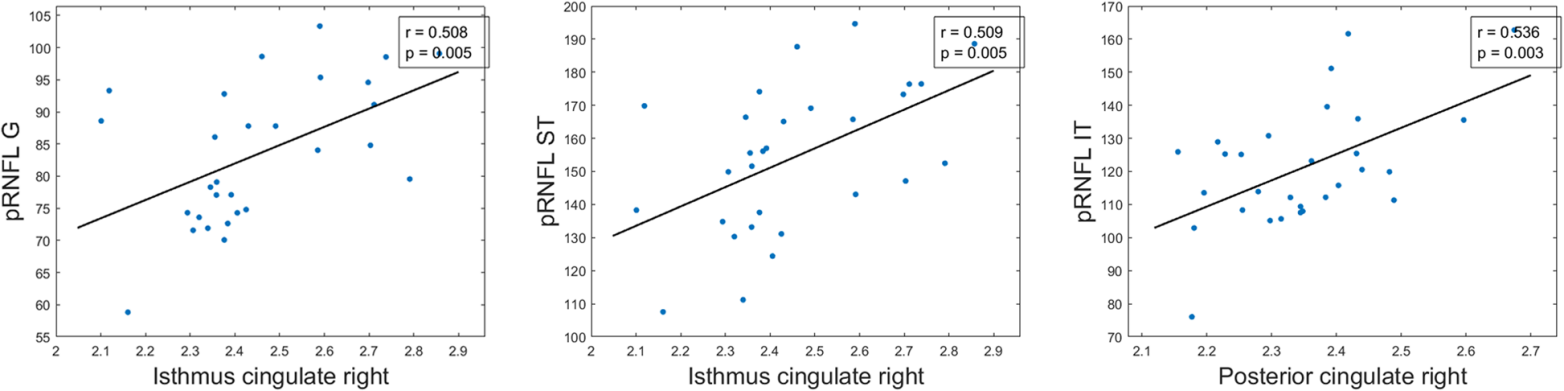
Scatter plots of some statistically significant age-adjusted correlations between peripapillary retinal nerve fiber layer thickness and volumes and thickness of brain structures in participants with high genetic risk of developing AD. pRNFL peripapillary retinal nerve fiber layer, G global, ST superotemporal, IT inferotemporal

**Fig. 6:**
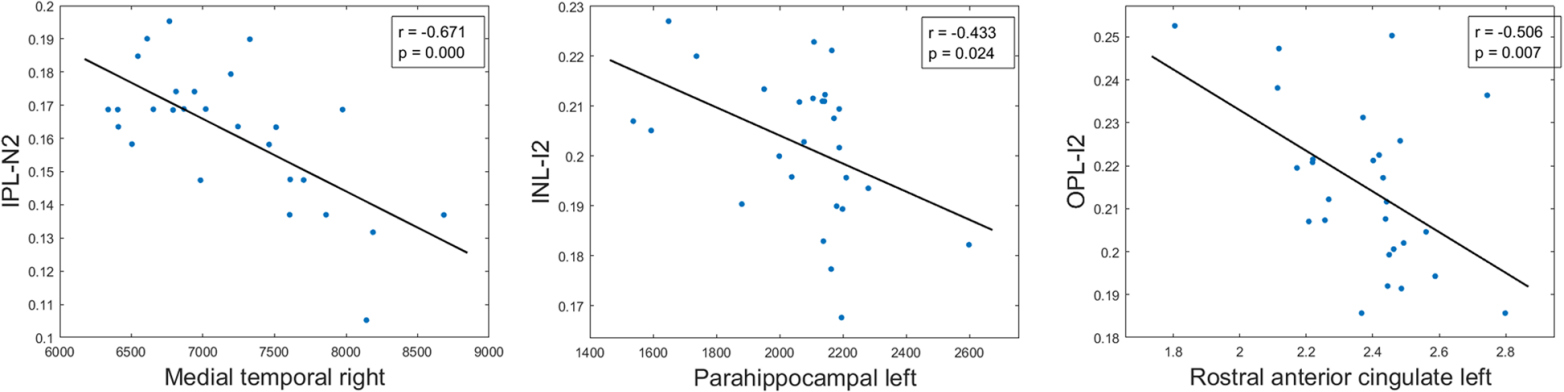
Scatter plots of some statistically significant age-adjusted correlations between retinal sector volumes and volumes and thickness of brain structures in participants without a high genetic risk of developing AD. IPL inner plexiform layer, N2 nasal sector of the outer macular ring, INL inner nuclear layer, I2 inferior sector of the outer macular ring, OPL outer plexiform layer

**Fig. 7:**
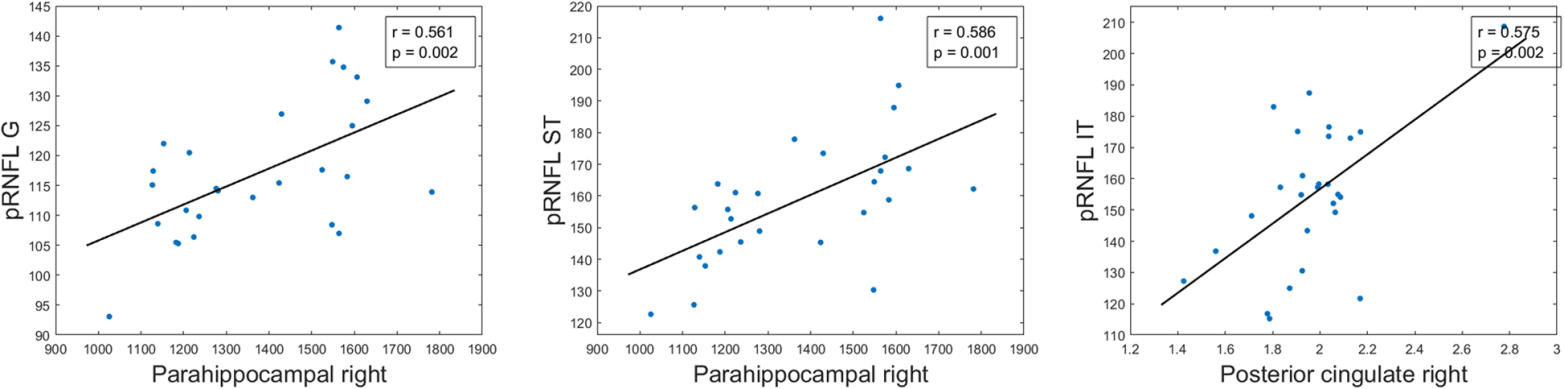
Scatter plots of some statistically significant age-adjusted correlations between peripapillary retinal nerve fiber layer thickness and volumes and thickness of brain structures in subjects with high genetic risk of developing AD. pRNFL peripapillary retinal nerve fiber layer, G global, ST superotemporal, IT inferotemporal

We highlighted correlations of brain MRI and OCT retinal volumes in those retinal sectors where we had found statistically significant retinal volume changes between the FH+ ApoE ɛ4+ and FH− ApoE ɛ4− groups.

### Correlations of participants with high genetic risk for developing AD

#### Macular sectors and brain correlations

We found that, in mRNFL, the foveal sector volume (C0) had a significant correlation (*p* < 0.05) with (i) the right isthmus cingulate (*r* = 0.390), (ii) the left lingual gyrus (*r*=0.446), (iii) left pericalcarine volume (*r* = 0.524), (iv) the right lateral occipital volume (*r* = 0.452), (v) the right cuneus volume (*r*=0.437), (vi) left cuneus volume (*r* = 0.547), (vii) the right occipital lobe (*r*=0.458), and (viii) the left occipital lobe (*r*=0.505) (Table 5) (Fig. 4).

In the OPL, the inferior sector (I2) of the outer macular ring was significantly associated (*p* < 0.05) with (i) right fusiforme thickness (*r* = 0.452), (ii) the right lingual gyrus (*r* = −0.380), (iii) right and left pericalcarine volume (*r* = −0.463 and −0.381, respectively), (iv) right and left cuneus volume (*r* = −0.486 and *r* = −0.485, respectively), and (v) right and left occipital lobe (*r*=−0.423 and *r*=−0.397, respectively) (Table 5 and Fig. 4).

#### pRNFL thickness and brain correlation

In terms of pRNFL thickness, we found significant correlations (*p* < 0.05) between retinal global value and (i) right caudal anterior cingulate thickness (*r* = 0.397), (ii) right posterior cingulate thickness (*r* = 0.423), and (iii) right and left isthmus cingulate thickness (*r* = 0.509 and *r* = 0.435, respectively) (Table 6 and Fig. 5).

The thickness of the temporal sector of pRNFL was only significant correlated with the thickness of the left posterior cingulate region (*r* = 0.405) and right lingual gyrus (*r*=0.422) (Table 6).

In pRNFL, the thickness of the superotemporal sector was significantly correlated (*p* < 0.05) with (i) right caudal anterior cingulate thickness (*r* = 0.413), (ii) right and left posterior cingulate thickness (*r* = 0.502 and *r* = 0.445), and (iii) right and left isthmus cingulate thickness (*r* = 0.509 and *r* = 0.414, respectively) (Table 6 and Fig. 5).

The thickness of the inferotemporal sector of pRNFL was significant associated with (i) right and left posterior cingulate thickness (*r* = 0.539 and *r* = 0.448, respectively), (ii) right and left isthmus cingulate thickness (*r* = 0.379 and *r*=0.434, respectively), and (iii) right ventral diencephalon volume (*r* = 0.381) (Table 6 and Fig. 5).

The thickness of the nasal sector of pRNFL was significantly correlated with the right entorhinal volume (*r* = −0.536) (Table 6).

In the pRNFL, superonasal thickness was significantly associated with (i) right isthmus cingulate thickness (*r* = 0.429) and (ii) left fusiform gyrus thickness (*r* = 0.412) (Table 6).

### Correlations in participants without genetic risk for developing AD

#### Macular sectors and brain correlations

In the IPL, we found an association between the outer macular ring in the nasal sector (N2) and (i) left parahippocampal volume (*r* = −0.429), (ii) right entorhinal volume (*r* = −0.516), (iii) right lingual gyrus volume (*r* = −0.395), (iv) left cuneus volume (*r* = −0.438), (v) right and left medial temporal lobe volume (*r* = −0.673 and *r* = −0.554, respectively), (vi) left amygdala volume (*r* = −0.501), (vii) right ventral diencephalon volume (*r* = −0.451), and (viii) right and left hippocampus volume (*r* = −0.542 and *r* = −0.595, respectively). In addition, in this layer, the volume of the inferior sector (I2) of the outer macular ring in was associated with (i) right entorhinal volume (*r* = −0.433), (ii) right medial temporal lobe volume (*r* = −0.534), (iii) right and left amygdala volume (*r* = −0.414 and *r* = −0.503, respectively), (iv) right ventral diencephalon volume (*r* = −0.412), and (v) right hippocampus volume (*r* = −0.394) (Table 7 and Fig. 6).

**Table 7:**
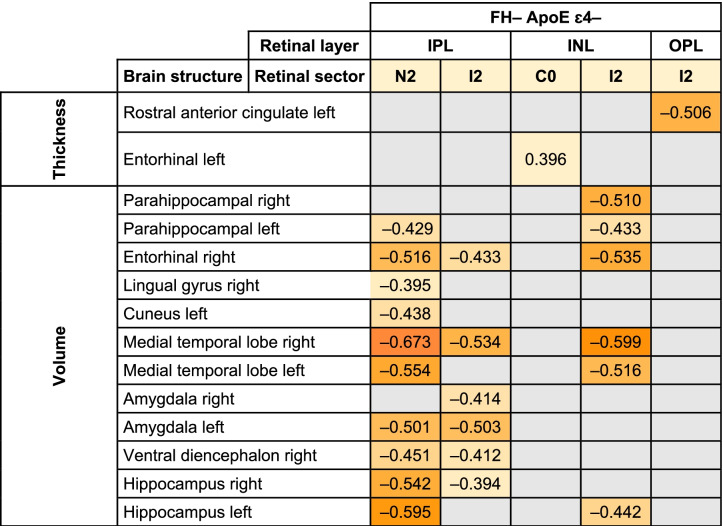
Significant age-adjusted correlations between retinal and brain structures in participants without a high genetic risk of developing AD. A colorimetric scale has been applied to the degree of correlation of the variables

*FH*– participants without a family history of AD, *ApoE ɛ4* noncarriers of ApoE, *IPL* inner plexiform layer, *INL* inner nuclear layer, *OPL* outer plexiform layer, *right* right hemisphere, and *left* left hemisphere

In the INL, the foveal macular sector (C0) was associated with left entorhinal thickness (*r* = −0.396). The inferior sector (I2) of the outer macular ring was associated with (i) right and left parahippocampal volume (*r* = −0.510 and *r* = −0.433, respectively), (ii) right entorhinal volume (*r* = −0.535), (iii) right and left medial temporal lobe volume (*r* = −0.599 and *r* = −0.516, respectively), and (iv) left hippocampus volume (*r* = −0.442) (Table 7 and Fig. 6).

Finally, in the OPL, the inferior sector (I2) of the outer macular ring was associated with left rostral anterior cingulate thickness (r = −0.506) (Table 7 and Fig. 6).

#### pRNFL thickness and brain correlation

In the group of participants without genetic risk for developing AD, we also found a significant correlation between global pRNFL thickness and right parahippocampal volume (*r* = 0.561) (Table 8 and Fig. 7).

**Table 8:**
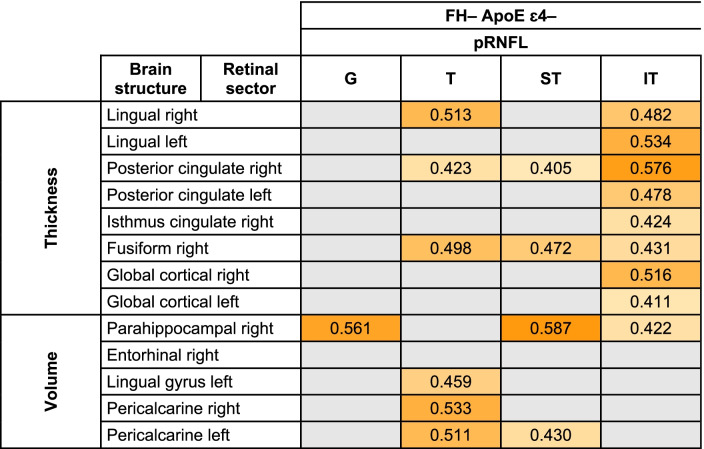
Significant age-adjusted correlations between pRNFL thickness in different sectors and thickness and volume of brain structures in participants without risk for developing AD. A colorimetric scale has been applied to the degree of correlation of the variables

*FH*– participants without family history of AD, *ApoE ɛ4–* noncarriers of ApoE ɛ4, *pRNFL* peripapillary retinal nerve fiber layer, *G* global, *T* temporal, *ST* superotemporal, *IT* inferotemporal, *right* right hemisphere; and *left* left hemisphere

The temporal sector of pRNFL had a significant correlation (*p* < 0.05) with (i) right lingual thickness (*r* = 0.513), (ii) right posterior cingulate thickness (*r* = 0.423), (iii) right fusiform thickness (*r* = 0.498), (iv) left lingual gyrus volume (*r* = 0.459), and (v) right and left pericalcarine volume (*r* = 0.533 and *r* = 0.511, respectively) (Table 8 and Fig. 7).

The thickness of the superotemporal sector of pRNFL was significantly associated with the (i) right posterior cingulate thickness (*r*=0.405), (ii) right fusiform thickness (*r* = 0.472), (iii) right parahippocampal volume (*r* = 0.587), and (iv) left pericalcarine volume (*r* = 0.430), while the inferotemporal thickness of pRNFL had a significant correlation with the (i) right and left lingual thickness (*r* = 0.482 and *r* = 0.534, respectively), (ii) right and left posterior cingulate thickness (*r* = 0.576 and *r* = 0.478, respectively), (iii) right cingulate isthmus thickness (*r* = 0.424), (iv) right fusiform thickness (*r* = 0.431), (v) right and left cortical thickness (*r* = 0.516 and *r* = 0.411, respectively), and (vi) right parahippocampal volume (*r* = 0.422) (Table 8 and Fig. 7).

## Discussion

To our knowledge, no previous study has studied the relationships between the retina and different brain structures in cognitively healthy participants but with two main genetic risk factors for developing the disease: (i) having a family history of sporadic senile form of AD and (ii) carrying at least one ɛ4 allele for the allele of the ApoE gene. One of the chief assets of this study was the careful selection of cases. Only family members of people diagnosed with sporadic senile AD were selected, and participants were free of ocular pathology and mental or cognitive disorders that could mask the results. We analyzed both macular and peripapillary areas of the retina in correlation with 20 brain structures, making this one of the studies that analyzed the correlation of the largest number of structures.

The higher number of female participants in the FH+ ApoE ɛ4+ group in comparison with male participants is due to the fact that women are more involved in caring for sick parents [26]. They are also more aware that participating in the studies will further advance the knowledge of Alzheimer’s disease to help both patients and their descendants [27].

Regarding the brain structural cortical thickness and volume, we did not find any correlation with the risk of developing an Alzheimer’s dementia based on familiar history or ApoE ɛ4 phenotype, except for the right cingulate isthmus, which almost reached a statistical difference between both study groups. Although this region is classically involved in AD degeneration, we cannot assess its significance as it is itself affected by all the other regions in our research. In the present study, a trend of a larger volume, without reaching statistical significance, was observed in different brain areas in the FH+ ApoE ɛ4+ group compared with the FH− ApoE ɛ4− group. Classically, it has been reported that subjects carrying at least one E4 allele for ApoE, throughout the temporal continuum of the disease and from several decades before, present a reduction of hippocampal volume [28–31] or a focal atrophy of this area [32, 33]. Nevertheless, an inflammatory reaction mediated by progranulin has been described in patients in early stages of the disease, who already present positive markers for amyloid, which also contributes to producing neuroinflammatory structural changes in preclinical stages of the disease [34].

From another point of view, our group has demonstrated previously that, when compared with the healthy control group, mild cognitive impairment patients exhibited a marked decrease in functional connectivity over posterior areas accompanied by an increased in anteriorventral regions of the brain, representing the common feature of the network failure starting in the pre-dementia stages of the disease, as a compensatory mechanism [35]. Although this increased connectivity has not been shown to require an increase in volume, it remains plausible that the increase in neuronal plasticity required to produce it would carry a transient physical increase in networks structures.

Both considering the compensatory or inflammatory hypothesis, a final possible explanation for this slight not significant increase in volume in our participants could be due to a statistical artifact being necessary to verify it in more extensive and longitudinal studies.

In our study, the FH+ ApoE ɛ4+ group showed a statistically significant volume decrease in the macular area in different retinal sectors compared with the FH− ApoE ɛ4− group. These results are in agreement with our previous study, in which we observed that there is a thinning of certain retinal sectors in relatives at high genetic risk for the development of AD [12].

It has been observed that family history of AD and the ApoE ɛ4 gene were associated with a thinning in the entorhinal cortex, subiculum, and medial temporal lobe, with these factors being additive to each other [18] and these structures the first to show signs of AD [36]. However, in our work, no statistically significant brain changes were observed between participants with FH+ ApoE ɛ4+ and FH− ApoE ɛ4−. This result is supported by previous studies that showed that retinal changes appear earlier than brain changes [13, 37–39]. Also, these retinal alterations, which could occur through retrograde transneuronal neurodegeneration [40], may be associated with atrophic brain changes already present before the appearance of clinical cognitive symptoms [17, 41].

The relationship between the brain and retina has been analyzed in other populations without a family history of AD and ApoE ɛ4 gene. There are previous studies that analyzed these correlations in normal older adults with mean ages of 68.0 ± 5.3 years, in which pRNFL thickness in the temporal quadrant was associated with temporal medial lobe volume and hippocampus volume, while the inferior quadrant was significantly associated with occipital lobe volume and selectively associated with the substructure of lingual gyrus volume [6]. In our work, in contrast to Shi et al., we did not find a correlation between the inferior quadrants of the pRNFL and the volume of the occipital lobe.

In a recent work undertaken in an older population (mean age: 65.1 ± 9.0 years), a positive correlation was observed between the pRNFL thickness and right and left hippocampal thickness [42]. Also, Méndez-Gómez et al., in an older population of 80.8 ± 3.9 years, found a direct correlation between pRNFL and the hippocampal fraction [43]. In agreement with these authors, we found a direct correlation in the FH+ ApoE ɛ4+ group between the thickness of the inferotemporal region of the pRNFL with (i) the thickness of right and left posterior cingulate; (ii) the right and left isthmus cingulate, being these, hippocampal areas; and (iii) the volume of right ventral diencephalon. The association between the retina and medial temporal lobe volume may indicate a degeneration in both tissues at the same time, preceding clinical cognitive changes in cognitively healthy participants at risk of AD [17].

In a 12-month longitudinal study, with participants with a mean age of 71.8 ± 3.9 years, an inverse association between the mean reduction in pRNFL thickness and the decrease in central cingulate cortex volume was observed. In addition, the reduction of pRNFL thickness in the inferior quadrants was associated with the decrease of central cingulate cortex volume [44]. We found the same correlation between inferotemporal pRNFL thickness with posterior cingulate thickness in our FH+ ApoE ɛ4+ participants. One possible explanation of the differences between previous studies and our work could be that our participants are young elder people with a mean age of 58.8 ± 6.0 years and we only accepted MMSE values > 26, whereas in Shi and colleagues’ studies, they accepted values for the Chinese version of the Mini-Mental State Examination (CMMSE) of ≥24. This issue is important, because normal cognitive values are above 26, and lower values could mask previous stages of the disease such as subjective memory complaints or mild cognitive impairment. Furthermore, we found no significant differences in pRNFL thickness between our study groups. Altered pRNFL thickness is known to be a good marker of disease progression [16]. On the other hand, changes in this retinal layer are associated with increased susceptibility to accumulation of neurofibrillary tangles and deposition of amyloid plaques in the occipital lobe and inferior temporal lobe, which are a part of the visual association cortex [45, 46].

Ong et al. observed, in a group of 164 patients aged 40–85 years including patients with cognitive impairment without dementia (*n* = 125), cognitively healthy participants (*n* = 36), and people with dementia (*n* = 3), that GCL-IPL thinning is associated with a reduction of gray matter in occipital and temporal lobe volume [47]. In another study performed on 79 neurologically normal adults with a mean age of 76.0 ± 5.5 years, the pRNFL reduction and the decline in total macular volume and GCL volume were significantly associated with a decrease in the medial temporal volumes of the hippocampus, the parahippocampal region, and the entorhinal region [17]. In a study carried out by Mutlu and colleagues, as part of the Rotterdam Study, older patients (67 ± 9.8 years old) with MMSE values of 28 ± 1.7, only the data of GCL, IPL, and pRNFL thickness measurements in relation to brain volume and hippocampal volume were analyzed [48]. In this study, it was found that the thinning of these retinal layers was significantly associated with lower brain volume and lower hippocampal volume, finding that this retinal thinning was associated with the thinning of both gray and white matter in the brain [48]. Chua et al., in a large population-based study (2131 participants) aged between 40 and 69 years old, also found that GCL thinning and total macular thickness were significantly associated with smaller hippocampal volume [49]. These findings are consistent with another study in 20 cognitively healthy, younger patients (50.5 ± 14.1 years old), where it was found that entorhinal gray matter volume was correlated with the GC-IPL complex [50]. In our FH− ApoE ɛ4− group, there was a significantly inverse correlation in the IPL between the volumes of the outer nasal macular sector and the volumes of the left parahippocampal region, the right entorhinal region, the right and left medial temporal lobule, the right and left hippocampus, the right lingual gyrus, the left cuneus, the right ventral diencephalon, and the left amygdala (Table 7). In addition, there was a significant inverse correlation in this retinal layer between the outer inferior macular sector and the right entorhinal, right medial temporal lobe, right ventral diencephalon, right hippocampus, and right and left amygdala (Table 7).

Carriers of the ApoE ɛ4 gene have a thinning of the entorhinal cortex in the left hemisphere compared with the right hemisphere, regardless of age or cognitive status [51]. In our FH− ApoE ɛ4− participants, there was a significant correlation between the left entorhinal thickness with the C0 retinal volume in the INL. Asymmetry of cerebral cortex thickness in the entorhinal region associate to the presence of an ApoE ɛ4 allele [52], may in itself reflect pathology, but could lead to future spatial and temporal distribution patterns of pathological changes [51]. This fact may explain, why in our participants with ApoE ɛ4+, the correlation between the nasal sector of the pRNFL with the right entorhinal volume was stronger than in ApoE ɛ4– participants.

As can be seen in the tables, most of the correlations found between the macular sectors and brain structures in FH− ApoE ɛ4− participants are inverse, while in participants with a high genetic risk for sporadic senile form of AD (FH+ ApoE ɛ4+), they were mostly direct. This could be due to the presence or not of the ApoE ɛ4 gene, as we have already seen in the retina [12], as well as to different behaviors in the central nervous system generated by the presence of this gene [51]. When the ApoE ɛ4 gene is present, changes could occur at the same time in the retina and in different brain areas, either due to the accumulation of Aβ or tau protein, or to structural brain changes that would be concomitant with retinal changes. However, the absence of the ApoE ɛ4 gene could generate different behavior as the correlations are inverse and a change in brain structures would not be accompanied by retinal changes, because they would not be related to it or would not be concomitant in their genesis.

### Limitations

Our study has strengths and limitations. The main strength of our work is that it is one of the brain–retina correlational studies that has analyzed the most brain areas, considering both right and left hemispheres. One of the limitations of our study is that our sample is small; however, we have made a strict selection of our participants: all participants at high genetic risk have a family history of AD and at least one ɛ4 allele for ApoE. In addition, all participants were cognitively healthy and had a MMSE score above 26. Furthermore, despite the small number of participants, the OCT results are already consistent, so if the sample was increased, certain MRI results, which are close to significance, would also be statistically significant. Longitudinal studies with larger samples of participants should be performed to confirm the etiopathogenic mechanisms involved in the changes occurring between the retina and the brain. Such studies are necessary to understand the evolution of imaging biomarkers as well as to better predict the possible establishment of the disease in participants at high genetic risk for the development of AD, so participants with mild cognitive impairment or more advanced stages of the disease should also be included. Furthermore, if our results were replicated by other groups, it would strengthen the interpretation of our findings on retina–brain correlations. In future studies, it would be necessary to include other biomarkers for AD such as cerebrospinal fluid analysis or positron emission tomography as they would increase the value of our findings [42].

## Conclusions

In conclusion, these results demonstrate that there is a correlation between changes in the retina and various brain structures in participants at high genetic risk for developing sporadic senile forms of AD. In these cognitively healthy participants, there is already a significant correlation between pRNFL thickness and the volume of brain areas closely related to AD such as the entorhinal cortex, the lingual gyrus, and the hippocampus. Moreover, with the recent approval by the Food and Drug Administration (FDA) of the first treatment capable of modifying the pathophysiology of AD, the search for cheap, non-invasive, and readily available biomarkers will be mandatory given the need for early diagnosis in participants at high risk of a pathology whose incidence will increase exponentially in the near future worldwide [53]. Therefore, OCT volume measurements and their correlations with brain area volumes could be a biomarker of AD, even in the preclinical stages of AD, and longitudinal studies are needed to really know how many of these participants eventually develop the disease.

## Supplementary Information

**Additional file 1: Table S1.** Significant age-adjusted Pearson correlations between macular volume of total retina and brain structures. **Table S2.** Significant age-adjusted Pearson correlations between macular RNFL and brain structures. **Table S3.** Significant age-adjusted Pearson correlations between GCL and brain structures. **Table S4.** Significant age-adjusted Pearson correlations between IPL and brain structures. **Table S5.** Significant age-adjusted Pearson correlations between INL and brain structures. **Table S6.** Significant age-adjusted Pearson correlations between OPL and brain structure. **Table S7.** Significant age-adjusted Pearson correlations between macular volume of ONL and brain structures. **Table S8.** Significant age-adjusted Pearson correlations between RPE and brain structures.

**Additional file 2: Figure S1.** Scatter plots of statistically significant correlations between retinal sector volumes and volumes and thickness of brain structures in participants with high genetic risk of developing AD.

**Additional file 3: Figure S2.** Scatter plots of statistically significant correlations between peripapillary retinal nerve fiber layer thickness and volumes and thickness of brain structures in participants with high genetic risk of developing AD.

**Additional file 4: Figure S3.** Scatter plots of statistically significant correlations between retinal sector volumes and volumes and thickness of brain structures in participants without a high genetic risk of developing AD.

**Additional file 5: Figure S4.** Scatter plots statistically significant correlations between peripapillary retinal nerve fiber layer thickness and volumes and thickness of brain structures in participants without a high genetic risk of developing AD.

## Data Availability

The datasets during and/or analyzed during the current study available from the corresponding author on reasonable request.

## Abbreviations

AD: Alzheimer’s disease
ApoE ɛ4: Apolipoprotein E4
Aβ: Amyloid-beta
C0: Central macular sector
CMMSE: Chinese version of the Mini-Mental State Examination
CSF: Cerebrospinal fluid
ETDRS: Early Treatment Diabetic Retinopathy Study
FDA: Food and Drug Administration
FH: Family history
G: Global
GCL: Ganglion cell layer
I1: Inferior sector of the inner macular ring
I2: Inferior sector of the outer macular ring
IN: Inferonasal
INL: Inner nuclear layer
IPL: Inner plexiform layer
IT: Inferotemporal
Lh: Left hemisphere
MMSE: Mini-Mental State Examination
MRI: Magnetic resonance imaging
mRNFL: Macular retinal nerve fiber layer
N: Nasal
N1: Nasal sector of the inner macular ring
N2: Nasal sector of the outer macular ring
OCT: Optical tomography coherence
ONL: Outer nuclear layer
OPL: Outer plexiform layer
PET: Positron emission tomography
pRNFL: Peripapillary retinal nerve fiber layer
Rh: Right hemisphere
RPE: Retinal pigment epithelium
S1: Superior sector of the inner macular ring
S2: Superior sector of the outer macular ring
SN: Superonasal
ST: Superotemporal
T: Temporal
T1: Temporal sector of the inner macular ring
T2: Temporal sector of the outer macular ring
