# Supplementary material for: The relationship between retinal layers and brain areas in asymptomatic first-degree relatives of sporadic forms of Alzheimer’s disease: an exploratory analysis
Source: Alzheimers Res Ther. 2022 Jun 4;14:79. doi: 10.1186/s13195-022-01008-5 (PMC9166601; doi:10.1186/s13195-022-01008-5)
Supplement: Supplementary file 1 — Additional file 1: Table S1. Significant age-adjusted Pearson correlations between macular volume of total retina and brain structures. Table S2. Significant age-adjusted Pearson correlations between macular RNFL and brain structures. Table S3. Significant age-adjusted Pearson correlations between GCL and brain structures. Table S4. Significant age-adjusted Pearson correlations between IPL and brain structures. Table S5. Significant age-adjusted Pearson correlations between INL and brain structures. Table S6. Significant age-adjusted Pearson correlations between OPL and brain structure. Table S7. Significant age-adjusted Pearson correlations between macular volume of ONL and brain structures. Table S8. Significant age-adjusted Pearson correlations between RPE and brain structures. [file 13195_2022_1008_MOESM1_ESM.docx]

**Supplementary table 1. Significant Pearson correlations between macular volume of total retina and brain structures.**

|  |  |  |  |  |  |  |  |  |  |  |  |  |  |  |  |  |  |  |  |  |
| --- | --- | --- | --- | --- | --- | --- | --- | --- | --- | --- | --- | --- | --- | --- | --- | --- | --- | --- | --- | --- |
|  |  | **Retinal layer** | **Total retina** | | | | | | | | | | | | | | | | | |
|  | **Retinal Sector** | **C0** | | **N1** | | **S1** | | **T1** | | **I1** | | **N2** | | **S2** | | **T2** | | **I2** | |  |
|  | **Brain structure** | **Study group** | **FH–ApoE ɛ4–** | **FH+ ApoE ɛ4+** | **FH–ApoE ɛ4–** | **FH+ ApoE ɛ4+** | **FH–ApoE ɛ4–** | **FH+ ApoE ɛ4+** | **FH–ApoE ɛ4–** | **FH+ ApoE ɛ4+** | **FH–ApoE ɛ4–** | **FH+ ApoE ɛ4+** | **FH– ApoE ɛ4–** | **FH+ ApoE ɛ4+** | **FH–ApoE ɛ4–** | **FH+ ApoE ɛ4+** | **FH–ApoE ɛ4–** | **FH+ ApoE ɛ4+** | **FH–ApoE ɛ4–** | **FH+ ApoE ɛ4+** |
| **Thickness** | Lingual right | | 0.435 |  |  |  |  |  | 0.413 |  |  |  |  |  |  |  | 0.432 |  | 0.416 |  |
|  | Lingual left | |  |  | 0.390 |  | 0.478 |  | 0.53 |  | 0.529 |  |  |  | 0.404 |  | 0.597 |  | 0.566 |  |
|  | Rostral anterior cingulate right | |  |  |  |  |  |  |  |  |  |  |  |  |  |  | 0.471 |  |  |  |
|  | Posterior cingulate right | |  |  | 0.406 |  | 0.418 |  | 0.447 |  | 0.430 |  |  |  |  |  | 0.492 |  | 0.420 |  |
|  | Isthmus cingulate right | |  |  |  |  |  |  |  |  |  |  |  |  |  |  |  |  | 0.431 |  |
|  | Entorhinal left | |  | 0.395 |  |  |  |  |  |  |  |  |  |  |  |  |  |  |  |  |
|  | Cortical thickness right | | 0.403 |  |  |  |  |  |  |  |  |  |  |  |  |  | 0.434 |  | 0.506 |  |
|  | Cortical thickness left | | 0.436 |  |  |  |  |  |  |  |  |  |  |  |  |  |  |  | 0.456 |  |
| **Volume** | Parahippocampal right | | 0.446 |  |  |  | 0.436 |  | 0.424 |  | 0.476 |  | 0.578 |  | 0.501 |  | 0.400 |  | 0.513 |  |
|  | Parahippocampal left | | 0.407 |  |  |  |  |  | 0.396 |  |  |  | 0.489 |  |  |  |  |  |  |  |
|  | Entorhinal right | | 0.417 | 0.476 |  | 0.494 |  |  |  | 0.439 |  | 0.398 |  |  |  |  |  |  |  |  |
|  | Entorhinal left | |  | 0.421 |  | 0.720 |  | 0.607 |  | 0.544 |  | 0.535 |  | 0.374 |  |  | 0.436 |  |  | 0.386 |
|  | Lingual girus left | |  |  |  |  |  |  |  |  |  |  |  |  |  |  |  |  |  |  |
|  | Pericalcarine right | |  |  |  |  |  |  |  |  |  |  |  | 0.404 |  |  | 0.472 |  | 0.405 | 0.415 |
|  | Pericalcarine left | | 0.440 |  |  |  | 0.412 |  |  |  | 0.416 |  |  |  | 0.471 |  | 0.497 |  | 0.525 |  |
|  | Lateral occipital left | |  |  |  |  |  | 0.393 |  |  |  |  |  |  |  |  |  |  |  |  |
|  | Cuneus right | | 0.458 |  |  |  |  |  |  |  |  |  |  |  |  |  | 0.452 |  | 0.410 |  |
|  | Cuneus left | | 0.689 |  |  |  | 0.456 |  | 0.490 |  | 0.508 |  |  |  |  |  | 0.484 |  | 0.461 |  |
|  | Medial temporal lobe right | | 0.595 | 0.375 |  | 0.464 |  | 0.388 |  | 0.452 |  |  |  |  |  |  |  |  |  |  |
|  | Medial temporal lobel left | | 0.483 |  |  | 0.441 |  | 0.392 |  |  |  |  |  |  |  |  |  |  |  |  |
|  | Occipital lobe right | |  |  |  |  |  |  |  |  |  |  |  |  |  |  | 0.416 |  |  |  |
|  | Occipital lobe left | |  |  |  |  |  |  | 0.39 |  |  |  |  |  |  |  | 0.461 |  | 0.427 |  |
|  | Ventral diencephalon right | | 0.498 |  |  |  |  |  |  |  |  |  |  |  |  |  |  |  |  |  |
|  | Ventral diencephalon left | |  |  |  | 0.385 |  |  |  |  |  |  |  |  |  |  |  |  |  |  |
|  | Hippocampus right | | 0.44 |  |  | 0.421 |  | 0.432 |  | 0.456 |  |  |  |  |  |  |  | 0.403 |  |  |
|  | Hippocampus left | | 0.479 |  |  |  |  |  |  |  |  |  |  |  |  |  |  |  |  |  |
| FH-: participants without a family history of AD; FH+: participants with a family history of AD; ApoE: Apolipoprotein E; right: right hemisphere and left: left hemisphere. C0: central macular sector; N1: nasal sector if the inner macular ring; I1: inferior sector of the inner macular ring; T1: temporal sector of the inner macular ring; S1: superior sector of the inner macular ring; N2: nasal sector of the outer macular ring. I2: inferior sector of the outer macular ring; T2: temporal sector of the outer macular ring; S2: superior sector of the outer macular ring; ST: supero-temporal; SN: supero-nasal; N: nasal; IN: infero-nasal; IT: infero-temporal; T: temporal | | | | | | | | | | | | | | | | | | | | |

**Supplementary table 2. Significant Pearson correlations between macular RNFL and brain structures.**

|  |  | **Retinal layer** | **RNFL** | | | | | | | | | | | | | | | | | |
| --- | --- | --- | --- | --- | --- | --- | --- | --- | --- | --- | --- | --- | --- | --- | --- | --- | --- | --- | --- | --- |
|  |  | **Retinal sector** | **C0** | | **N1** | | **S1** | | **T1** | | **I1** | | **N2** | | **S2** | | **T2** | | **I2** | |
|  | **Brain area** | **Study group** | **FH–ApoE ɛ4–** | **FH+ ApoE ɛ4+** | **FH–ApoE ɛ4–** | **FH+ ApoE ɛ4+** | **FH– ApoE ɛ4–** | **FH+ ApoE ɛ4+** | **FH–ApoE ɛ4–** | **FH+ ApoE ɛ4+** | **FH– ApoE ɛ4–** | **FH+ ApoE ɛ4+** | **FH–ApoE ɛ4–** | **FH+ ApoE ɛ4+** | **FH–ApoE ɛ4–** | **FH+ ApoE ɛ4+** | **FH– ApoE ɛ4–** | **FH+ ApoE ɛ4+** | **FH–ApoE ɛ4–** | **FH+ ApoE ɛ4+** |
|  | Lingual right | |  |  | **0.401** |  |  |  |  |  |  |  |  |  |  |  |  |  |  |  |
| **Thickness** | Lingual left | |  |  |  |  |  |  |  |  |  |  |  |  |  | -0.41 |  |  |  |  |
|  | Rostral anterior cingulate right | |  |  |  |  |  |  |  |  | -0.452 |  |  |  |  |  |  |  |  |  |
|  | Rostral anterior cingulate left | |  |  |  |  |  |  |  |  |  |  |  |  | -0.612 |  |  |  |  |  |
|  | Caudal anterior cingulate left | |  |  |  |  |  |  | -0.576 |  |  |  |  |  | -0.416 |  |  |  |  |  |
|  | Posterior cingulate right | |  |  |  |  |  |  |  |  |  |  |  |  |  |  |  |  |  |  |
|  | Posterior cingulate left | |  |  |  |  | -0.44 |  |  |  |  |  |  |  |  |  |  | -0.388 |  |  |
|  | Isthmus cingulate right | |  | 0.390 |  |  |  |  |  |  |  |  |  |  |  |  |  |  |  |  |
|  | Entorhinal right | |  |  |  |  |  |  | -0.538 |  |  |  |  |  |  |  |  | 0.463 |  |  |
|  | Entorhinal left | |  |  |  |  |  |  |  |  |  |  |  |  |  |  |  |  |  |  |
|  | Fusiform right | |  |  |  |  |  |  | -0.43 |  |  |  |  |  |  |  |  |  |  |  |
|  | Fusiform left | |  |  |  |  | -0.525 |  | -0.395 |  |  |  |  |  |  |  | -0.447 |  |  |  |
| **Volume** | Parahippocampal right | |  |  |  |  |  |  |  |  | -0.392 |  |  |  |  |  |  |  |  |  |
|  | Parahippocampal left | |  |  |  |  |  |  |  |  |  |  |  |  | -0.409 |  |  |  | -0.442 |  |
|  | Volumen lingual gyrus left | |  | 0.446 |  |  |  |  |  |  |  |  |  |  |  |  |  |  |  |  |
|  | Pericalcarine right | |  |  |  |  |  |  |  |  |  |  |  |  |  |  |  |  |  |  |
|  | Pericalcarine left | |  | 0.524 |  |  |  |  |  |  |  |  |  |  |  |  |  |  |  |  |
|  | Lateral occipital right | |  | 0.452 |  |  |  | -0.435 |  |  |  |  |  |  |  |  |  |  |  |  |
|  | Cuneus right | |  | 0.437 |  |  |  |  |  |  |  |  |  |  |  |  |  |  |  |  |
|  | Cuneus left | |  | 0.547 |  |  |  |  |  |  |  |  |  |  |  |  |  |  |  |  |
|  | Temporal medial lobe right | |  |  |  |  |  | -0.419 |  |  |  |  |  |  |  |  |  |  |  |  |
|  | Occipital lobe right | |  | 0.458 |  |  |  | -0.388 |  |  |  |  |  |  |  |  |  |  |  |  |
|  | Occipital lobe left | |  | 0.505 |  |  |  |  |  |  |  |  |  |  |  |  |  |  |  |  |
|  | Amygdala right | |  |  | 0.404 |  |  |  |  |  |  |  |  |  |  |  |  |  |  |  |
|  | Amygdala left | |  |  |  |  |  |  |  |  |  |  |  |  |  | -0.451 |  |  |  |  |
|  | Hippocampus right | |  |  |  |  |  | -0.412 |  |  |  | -0.427 |  | -0.391 |  |  |  |  |  |  |
|  | Intracranial |  |  |  |  | -0.485 |  |  |  |  |  |  |  |  |  |  |  |  |  |  |
|  | | | | | | | | | | | | | | | | | | | | |
| FH-: participants without a family history of AD; FH+: participants with a family history of AD; ApoE: Apolipoprotein E; right: right hemisphere and left: left hemisphere. C0: central macular sector; N1: nasal sector if the inner macular ring; I1: inferior sector of the inner macular ring; T1: temporal sector of the inner macular ring; S1: superior sector of the inner macular ring; N2: nasal sector of the outer macular ring, I2: inferior sector of the outer macular ring; T2: temporal sector of the outer macular ring; S2: superior sector of the outer macular ring; ST: supero-temporal; SN: supero-nasal; N: nasal; IN: infero-nasal; IT: infero-temporal; T: temporal | | | | | | | | | | | | | | | | | | | | |
|  | | | | | | | | | | | | | | | | | | | | |

**Supplementary table 3. Significant Pearson correlations between GCL and brain structures.**

|  |  | **Retinal layer** | **GCL** | | | | | | | | | | | | | | | | | |
| --- | --- | --- | --- | --- | --- | --- | --- | --- | --- | --- | --- | --- | --- | --- | --- | --- | --- | --- | --- | --- |
|  | **Retinal sector** | **C0** | | **N1** | | **S1** | | **T1** | | **I1** | | **N2** | | **S2** | | **T2** | | **I2** | |  |
|  | **Brain Structure** | **Study groups** | **FH–ApoE ɛ4–** | **FH+ ApoE ɛ4+** | **FH– ApoE ɛ4–** | **FH+ ApoE ɛ4+** | **FH– ApoE ɛ4–** | **FH+ ApoE ɛ4+** | **FH–ApoE ɛ4–** | **FH+ ApoE ɛ4+** | **FH–ApoE ɛ4–** | **FH+ ApoE ɛ4+** | **FH–ApoE ɛ4–** | **FH+ ApoE ɛ4+** | **FH– ApoE ɛ4–** | **FH+ ApoE ɛ4+** | **FH–ApoE ɛ4–** | **FH+ ApoE ɛ4+** | **FH– ApoE ɛ4–** | **FH+ ApoE ɛ4+** |
| **Thickness** | Posterior cingulate right | |  |  |  |  |  |  |  |  |  |  |  | -0.395 |  |  |  |  |  |  |
|  | Posterior cingulate left | |  |  |  |  |  |  |  |  |  |  |  |  |  |  |  |  |  |  |
| **Volume** | Parahippocampal right | |  |  |  |  |  |  |  |  |  |  |  |  |  |  | -0.408 |  |  |  |
|  | Parahippocampal left | |  |  |  |  |  |  |  |  |  |  |  |  |  |  | -0.393 |  |  |  |
|  | Entorhinal right | |  |  |  |  |  |  |  |  |  |  | -0.482 |  |  |  | -0.394 |  | -0.446 |  |
|  | Lingual girus right | |  |  |  |  |  |  |  |  |  |  | -0.421 |  | -0.479 |  |  |  |  |  |
|  | Cuneus right | |  |  |  |  |  |  |  |  |  |  |  |  |  |  |  |  |  |  |
|  | Cuneus left | |  |  |  |  |  |  |  |  |  |  | -0.439 |  |  |  |  |  |  |  |
|  | Temporal medial lobe right | |  |  |  |  |  |  |  |  |  |  | -0.592 |  | -0.495 |  | -0.446 |  | -0.536 |  |
|  | Temporal medial lobe left | |  |  |  |  |  |  |  |  |  |  | -0.467 |  | -0.51 |  | -0.487 |  |  |  |
|  | Amygdala right | |  |  |  |  |  |  |  |  |  |  |  |  | -0.415 |  | -0.389 |  |  |  |
|  | Amygdala left | |  |  | -0.391 |  | -0.443 |  |  |  |  |  | -0.503 |  | -0.521 |  |  |  | -0.523 |  |
|  | Ventral diencephalon right | |  | 0.407 |  |  |  |  |  |  |  |  | -0.401 |  | -0.427 |  |  |  |  | 0.392 |
|  | Ventral diencephalon left | |  |  |  |  |  |  |  |  |  |  |  |  |  |  |  |  | -0.422 |  |
|  | Hippocampus right | |  |  |  |  |  |  |  |  |  |  | -0.477 |  |  |  |  |  | -0.424 |  |
|  | Hippocampus left | |  |  |  |  |  |  |  |  |  |  | -0.49 |  | -0.487 |  | -0.454 |  |  |  |
|  | Intracraneal | |  |  |  |  |  |  |  |  |  |  |  |  |  |  |  |  |  |  |
|  | | | | | | | | | | | | | | | | | | | | |
| FH-: participants without a family history of AD; FH+: participants with a family history of AD; ApoE: Apolipoprotein E; right: right hemisphere and left: left hemisphere, C0: central macular sector; N1: nasal sector if the inner macular ring; I1: inferior sector of the inner macular ring; T1: temporal sector of the inner macular ring; S1: superior sector of the inner macular ring; N2: nasal sector of the outer macular ring, I2: inferior sector of the outer macular ring; T2: temporal sector of the outer macular ring; S2: superior sector of the outer macular ring; ST: supero-temporal; SN: supero-nasal; N: nasal; IN: infero-nasal; IT: infero-temporal; T: temporal | | | | | | | | | | | | | | | | | | | | |

**Supplementary table 4. Significant Pearson correlations between IPL and brain structures.**

|  |  | **Retinal layer** | **IPL** | | | | | | | | | | | | | | | | | |
| --- | --- | --- | --- | --- | --- | --- | --- | --- | --- | --- | --- | --- | --- | --- | --- | --- | --- | --- | --- | --- |
|  |  | **Retinal sector** | **C0** | | **N1** | | **S1** | | **T1** | | **I1** | | **N2** | | **S2** | | **T2** | | **I2** | |
| **Brain structure** | **Study groups** | **FH–ApoE ɛ4–** | **FH+ ApoE ɛ4+** | **FH–ApoE ɛ4–** | **FH+ ApoE ɛ4+** | **FH–ApoE ɛ4–** | **FH+ ApoE ɛ4+** | **FH– ApoE ɛ4–** | **FH+ ApoE ɛ4+** | **FH– ApoE ɛ4–** | **FH+ ApoE ɛ4+** | **FH– ApoE ɛ4–** | **FH+ ApoE ɛ4+** | **FH– ApoE ɛ4–** | **FH+ ApoE ɛ4+** | **FH–ApoE ɛ4–** | **FH+ ApoE ɛ4+** | **FH–ApoE ɛ4–** | **FH+ ApoE ɛ4+** |  |
| **Volume** | Parahippocampal right | |  |  |  |  |  |  |  |  |  |  |  |  | -0.427 |  | -0.488 |  |  |  |
|  | Parahippocampal left | |  |  |  |  |  |  |  | 0.403 |  |  | -0.429 |  | -0.438 |  |  |  |  |  |
|  | Entorhinal right | |  |  |  |  |  |  |  |  |  |  | -0.516 |  | -0.47 |  | -0.478 |  | -0.433 |  |
|  | Entorhinal left | |  |  |  |  |  |  |  |  |  |  |  |  |  |  |  |  |  |  |
|  | Lingual gyrus right | |  |  |  |  |  |  |  |  |  |  | -0.395 |  | -0.429 |  | -0.432 |  |  |  |
|  | Cuneus left | |  |  |  |  |  |  |  |  |  |  | -0.438 |  | -0.469 |  |  |  |  |  |
|  | Temporal medial lobe right | |  |  |  |  |  |  |  |  |  |  | -0.673 |  | -0.591 |  | -0.505 |  | -0.534 |  |
|  | Temporal medial lobe left | |  |  |  |  |  |  |  |  |  |  | -0.554 |  | -0.556 |  | -0.438 |  |  |  |
|  | Amygdala right | |  |  |  |  |  |  |  |  |  |  |  |  |  |  |  |  | -0.414 |  |
|  | Amygdala left | |  |  |  |  |  |  |  |  |  |  | -0.501 |  | -0.442 |  |  |  | -0.503 |  |
|  | Ventral diencephalon right | |  | 0.498 |  |  |  |  |  |  |  |  | -0.451 |  |  |  |  |  | -0.412 |  |
|  | Ventral diencephalon left | |  |  |  |  |  |  |  |  |  |  |  |  |  |  |  |  |  |  |
|  | Hippocampus right | |  |  |  |  |  |  |  |  |  |  | -0.542 |  | -0.402 |  |  |  | -0.394 |  |
|  | Hippocampus left | |  |  |  |  |  |  |  |  |  |  | -0.595 |  | -0.517 |  | -0.420 |  |  |  |
|  | | | | | | | | | | | | | | | | | | | | |
| FH-: participants without a family history of AD; FH+: participants with a family history of AD; ApoE: Apolipoprotein E; right: right hemisphere and left: left hemisphere. C0: central macular sector; N1: nasal sector in the inner macular ring; I1: inferior sector of the inner macular ring; T1: temporal sector of the inner macular ring; S1: superior sector of the inner macular ring; N2: nasal sector of the outer macular ring. I2: inferior sector of the outer macular ring; T2: temporal sector of the outer macular ring; S2: superior sector of the outer macular ring; ST: supero-temporal; SN: supero-nasal; N: nasal; IN: infero-nasal; IT: infero-temporal; T: temporal | | | | | | | | | | | | | | | | | | | | |
|  | | | | | | | | | | | | | | | | | | | | |

**Supplementary table 5. Significant Pearson correlations between INL and brain structures.**

|  |  | **Retinal layer** | **INL** | | | | | | | | | | | | | | | | | |
| --- | --- | --- | --- | --- | --- | --- | --- | --- | --- | --- | --- | --- | --- | --- | --- | --- | --- | --- | --- | --- |
|  | **Retinal sector** | **C0** | | **N1** | | **S1** | | **T1** | | **I1** | | **N2** | | **S2** | | **T2** | | **I2** | |  |
|  | **Brain structure** | **Study groups** | **FH–ApoE ɛ4–** | **FH+ ApoE ɛ4+** | **FH–ApoE ɛ4–** | **FH+ ApoE ɛ4+** | **FH–ApoE ɛ4–** | **FH+ ApoE ɛ4+** | **FH–ApoE ɛ4–** | **FH+ ApoE ɛ4+** | **FH–ApoE ɛ4–** | **FH+ ApoE ɛ4+** | **FH–ApoE ɛ4–** | **FH+ ApoE ɛ4+** | **FH–ApoE ɛ4–** | **FH+ ApoE ɛ4+** | **FH–ApoE ɛ4–** | **FH+ ApoE ɛ4+** | **FH–ApoE ɛ4–** | **FH+ ApoE ɛ4+** |
| **Thickness** | Rostral anterior cingulate right | |  |  |  |  |  |  |  |  |  |  |  |  |  |  |  |  |  |  |
|  | Rostral anterior cingulate left | |  |  |  |  |  |  |  |  |  |  |  |  |  |  |  |  |  |  |
|  | Caudal anterior cingulate right | |  |  |  |  |  |  |  |  |  |  |  |  |  |  |  |  |  |  |
|  | Caudal anterior cingulate left | |  |  |  |  |  |  |  |  |  |  | -0.400 |  |  |  | -0.437 |  |  |  |
|  | Isthmus cingulate left | |  |  |  |  |  | -0.501 |  |  |  |  |  |  |  |  |  |  |  |  |
|  | Entorhinal right | |  |  |  |  |  |  |  |  |  |  |  |  |  |  |  | -0.391 |  |  |
|  | Entorhinal left | | 0.396 |  |  |  |  |  |  |  |  |  |  |  |  |  |  |  |  |  |
|  | Fusiform right | |  |  | -0.450 |  |  |  |  |  |  |  |  |  |  |  |  |  |  |  |
|  | Fusiform left | |  |  |  |  |  |  |  |  |  |  |  |  |  |  |  |  |  |  |
| **Volume** | Parahippocampal right | |  |  | -0.574 |  | -0.425 |  | -0.405 |  | -0.451 |  |  |  |  |  | -0.466 |  | -0.510 |  |
|  | Parahippocampal left | |  |  |  |  |  |  |  |  |  |  | -0.431 |  |  |  | -0.438 |  | -0.433 |  |
|  | Entorhinal right | |  |  |  |  |  |  |  |  |  |  | -0.510 |  | -0.505 |  | -0.607 |  | -0.535 |  |
|  | Entorhinal left | |  |  |  |  |  |  |  |  |  |  | -0.405 |  | -0.404 |  | -0.540 |  |  |  |
|  | Lingual gyrus right | |  |  | -0.554 |  | -0.451 |  |  |  |  |  | -0.460 |  | -0.442 |  | -0.442 |  |  |  |
|  | Lingual gyrus left | |  |  | -0.482 |  |  |  |  |  | -0.489 | -0.412 |  |  |  |  |  |  |  |  |
|  | Pericalcarine left | |  |  |  |  |  |  |  |  |  |  |  |  |  |  |  |  |  |  |
|  | Cuneus right | |  |  |  |  |  |  |  |  |  | -0.392 |  |  |  |  |  |  |  |  |
|  | Cuneus left | |  |  |  |  | -0.392 |  |  |  |  |  |  |  |  |  |  |  |  |  |
|  | Medial temporal lobe right | |  |  | -0.517 |  | -0.451 |  | -0.411 |  | -0.402 |  | -0.611 |  | -0.571 |  | -0.662 |  | -0.559 |  |
|  | Medial temporal lobe left | |  |  | -0.474 |  |  |  |  |  |  |  | -0.566 |  | -0.468 |  | -0.629 |  | -0.516 |  |
|  | Occipital lobe right | |  |  |  |  |  |  |  |  |  |  |  |  |  |  | -0.397 |  |  |  |
|  | Occipital lobe left | |  |  |  |  |  |  |  |  |  | -0.397 |  |  |  |  |  |  |  |  |
|  | Amygdala right | |  |  |  |  |  |  |  |  |  | -0.409 |  |  |  |  |  |  |  |  |
|  | Amygdala left | |  |  |  |  |  |  |  |  |  |  | -0.415 |  |  |  | -0.435 |  |  |  |
|  | Ventral diencephalon right | |  |  | -0.413 | 0.464 |  |  |  |  |  |  | -0.430 |  | -0.455 |  | -0.537 |  |  |  |
|  | Ventral diencephalon left | |  |  |  | 0.392 |  |  |  |  |  |  |  |  |  |  | -0.499 |  |  |  |
|  | Hippocampus right | |  |  |  |  |  |  |  |  |  |  | -0.442 |  | -0.416 |  | -0.394 |  |  |  |
|  | Hippocampus left | |  |  | -0.535 |  |  |  |  |  |  |  | -0.522 |  | -0.401 |  | -0.522 |  | -0.442 |  |
|  | Intracraneal | |  |  |  |  |  |  |  |  |  |  |  |  |  |  |  |  |  |  |
|  | | | | | | | | | | | | | | | | | | | | |
| FH-: participants without a family history of AD; FH+: participants with a family history of AD; ApoE: Apolipoprotein E; right: right hemisphere and left: left hemisphere. C0: central macular sector; N1: nasal sector if the inner macular ring; I1: inferior sector of the inner macular ring; T1: temporal sector of the inner macular ring; S1: superior sector of the inner macular ring; N2: nasal sector of the outer macular ring. I2: inferior sector of the outer macular ring; T2: temporal sector of the outer macular ring; S2: superior sector of the outer macular ring; ST: supero-temporal; SN: supero-nasal; N: nasal; IN: infero-nasal; IT: infero-temporal; T: temporal | | | | | | | | | | | | | | | | | | | | |
|  | | | | | | | | | | | | | | | | | | | | |

**Supplementary table 6. Significant Pearson correlations between OPL and brain structure.**

|  |  |  |  |  |  |  |  |  |  |  |  |  |  |  |  |  |  |  |  |  |
| --- | --- | --- | --- | --- | --- | --- | --- | --- | --- | --- | --- | --- | --- | --- | --- | --- | --- | --- | --- | --- |
|  |  | **Retinal layer** | **OPL** | | | | | | | | | | | | | | | | | |
|  | **Retinal sector** | **C0** | | **N1** | | **S1** | | **T1** | | **I1** | | **N2** | | **S2** | | **T2** | | **I2** | |  |
|  | **Brain structure** | **Study groups** | **FH–ApoE ɛ4–** | **FH+ ApoE ɛ4+** | **FH–ApoE ɛ4–** | **FH+ ApoE ɛ4+** | **FH– ApoE ɛ4–** | **FH+ ApoE ɛ4+** | **FH–ApoE ɛ4–** | **FH+ ApoE ɛ4+** | **FH–ApoE ɛ4–** | **FH+ ApoE ɛ4+** | **FH– ApoE ɛ4–** | **FH+ ApoE ɛ4+** | **FH– ApoE ɛ4–** | **FH+ ApoE ɛ4+** | **FH– ApoE ɛ4–** | **FH+ ApoE ɛ4+** | **FH–ApoE ɛ4–** | **FH+ ApoE ɛ4+** |
| **Thickness** | Lingual right | |  |  |  |  |  |  |  |  |  | 0.416 | -0.405 |  |  |  |  |  |  |  |
|  | Rostral anterior cingulate left | |  |  |  |  |  |  |  |  | -0.532 |  |  |  |  |  | -0.594 |  | -0.506 |  |
|  | Isthmus cingulate right | |  |  |  |  |  |  |  |  |  |  | -0.427 |  |  |  |  |  |  |  |
|  | Isthsmus cingulate left | |  |  |  |  |  |  |  |  |  |  |  |  |  | -0.467 |  |  |  |  |
|  | Entorhinal right | | 0.435 |  |  |  |  |  |  |  |  |  |  |  |  |  |  |  |  |  |
|  | Fusiform rh | |  |  |  |  |  |  |  |  |  | 0.479 | -0.496 |  | -0.4 |  |  |  |  | 0.452 |
|  | Fusiform left | |  |  |  |  |  |  |  |  |  | 0.401 |  |  |  |  |  |  |  |  |
|  | Cortical thickness right | |  |  |  |  |  | -0.454 |  |  |  | 0.531 |  |  |  | -0.422 |  |  |  |  |
|  | Cortical thickness left | | 0.389 |  |  |  |  | -0.465 |  |  |  | 0.514 |  |  |  | -0.419 |  |  |  |  |
| **Volume** | Parahippocampal right | |  |  |  |  |  |  |  |  |  |  |  |  |  |  |  |  |  |  |
|  | Lingual gyrus right | |  |  |  |  |  |  |  |  |  |  |  |  |  |  |  |  |  | -0.38 |
|  | Pericalcarine right | |  |  |  |  |  |  |  |  |  |  |  |  |  |  |  |  |  | -0.463 |
|  | Pericalcarine left | |  |  |  |  |  |  |  |  |  |  |  |  |  |  |  |  |  | -0.381 |
|  | Cuneus right | |  |  |  |  |  |  |  |  |  |  |  |  |  |  |  |  |  | -0.486 |
|  | Cuneus left | |  |  |  |  |  |  |  |  |  |  |  | -0.422 |  |  |  | -0.378 |  | -0.485 |
|  | Occipital lobe right | |  |  |  |  |  |  |  |  |  |  |  |  |  |  |  |  |  | -0.423 |
|  | Occipital lobe left | |  |  |  |  |  |  |  |  |  |  |  |  |  |  |  |  |  | -0.397 |
|  | Amygdala right | | -0.406 |  |  |  |  |  |  |  |  |  |  |  |  |  |  |  |  |  |
|  | Ventral diencephalon right | |  |  |  |  |  |  |  |  |  |  |  |  |  |  |  |  | -0.389 |  |
|  | Hippocampus right | |  |  |  | 0.502 |  |  |  |  |  |  | -0.43 |  |  |  |  |  |  |  |
|  | Hippocampus left | |  |  | -0.517 |  |  |  |  |  |  |  | -0.475 |  |  |  |  |  |  |  |
|  | | | | | | | | | | | | | | | | | | | | |
| FH-: participants without a family history of AD; FH+: participants with a family history of AD; ApoE: Apolipoprotein E; right: right hemisphere and left: left hemisphere. C0: central macular sector; N1: nasal sector if the inner macular ring; I1: inferior sector of the inner macular ring; T1: temporal sector of the inner macular ring; S1: superior sector of the inner macular ring; N2: nasal sector of the outer macular ring. I2: inferior sector of the outer macular ring; T2: temporal sector of the outer macular ring; S2: superior sector of the outer macular ring; ST: supero-temporal; SN: supero-nasal; N: nasal; IN: infero-nasal; IT: infero-temporal; T: temporal | | | | | | | | | | | | | | | | | | | | |

**Supplementary table 7. Significant Pearson correlations between macular volume of ONL and brain structures.**

|  |  | **Retinal layer** | **ONL** | | | | | | | | | | | | | | | | | |
| --- | --- | --- | --- | --- | --- | --- | --- | --- | --- | --- | --- | --- | --- | --- | --- | --- | --- | --- | --- | --- |
|  |  | **Retinal sector** | **C0** | | **N1** | | **S1** | | **T1** | | **I1** | | **N2** | | **S2** | | **T2** | | **I2** | |
|  | **Brain structure** | **Study groups** | **FH– ApoE ɛ4–** | **FH+ ApoE ɛ4+** | **FH– ApoE ɛ4–** | **FH+ ApoE ɛ4+** | **FH– ApoE ɛ4–** | **FH+ ApoE ɛ4+** | **FH– ApoE ɛ4–** | **FH+ ApoE ɛ4+** | **FH–ApoE ɛ4–** | **FH+ ApoE ɛ4+** | **FH–ApoE ɛ4–** | **FH+ ApoE ɛ4+** | **FH–ApoE ɛ4–** | **FH+ ApoE ɛ4+** | **FH–ApoE ɛ4–** | **FH+ ApoE ɛ4+** | **FH– ApoE ɛ4–** | **FH+ ApoE ɛ4+** |
| **Thickness** | Lingual right | |  |  |  | -0.389 |  |  |  |  |  | -0.448 |  | -0.464 |  |  |  |  |  | -0.440 |
|  | Rostral anterior cingulate right | |  |  |  |  |  |  | -0.428 |  |  |  |  |  |  |  |  |  |  |  |
|  | Rostral anterior cingulate left | |  |  |  |  |  |  |  |  |  |  |  |  |  |  |  |  |  |  |
|  | Isthmus cingulate right | |  |  |  |  |  |  |  |  |  |  |  |  |  |  |  |  |  |  |
|  | Isthmus cingulate left | |  |  |  |  |  | 0.452 |  |  |  |  |  |  |  | 0.496 |  |  |  |  |
|  | Fusiform right | | -0.392 |  |  |  |  |  |  |  |  |  |  |  |  |  |  |  |  |  |
|  | Fusiform left | |  |  |  |  |  |  |  |  |  | -0.412 |  |  |  |  |  |  |  | -0.387 |
|  | Global cortical right | |  |  |  |  |  |  |  |  |  | -0.445 |  |  |  |  |  |  |  | -0.392 |
|  | Global cortical left | |  |  |  |  |  |  |  |  |  | -0.463 |  |  |  |  |  |  |  | -0.396 |
| **Volume** | Parahippocampal right | |  |  |  |  |  |  |  |  |  |  |  | 0.385 |  | 0.395 | -0.415 |  |  |  |
|  | Parahippocampal left | |  |  |  |  |  |  |  |  |  |  |  |  |  | 0.384 |  |  |  |  |
|  | Lingual gyrus right | |  |  |  |  |  |  |  |  |  |  |  |  |  |  | -0.451 |  |  |  |
|  | Pericalcarine right | |  |  |  |  |  |  |  |  |  |  |  |  |  |  | -0.443 |  |  |  |
|  | Pericalcarine left | |  |  |  |  |  |  |  |  |  |  |  |  |  |  | -0.459 |  |  |  |
|  | Lateral occipital right | |  |  |  |  |  |  |  |  |  | 0.390 |  |  |  |  |  |  |  | 0.423 |
|  | Temporal medial lobe left | |  |  |  |  |  |  |  |  |  |  |  |  |  |  |  |  |  |  |
|  | Amygdala | |  |  |  |  |  |  |  |  |  |  |  |  |  |  |  | 0.380 |  |  |
|  | Hippocampus left | |  |  | 0.557 |  |  |  |  |  |  |  |  |  |  |  |  |  |  |  |
|  | | | | | | | | | | | | | | | | | | | | |
| FH-: participants without a family history of AD; FH+: participants with a family history of AD; ApoE: Apolipoprotein E; right: right hemisphere and left: left hemisphere. C0: central macular sector; N1: nasal sector if the inner macular ring; I1: inferior sector of the inner macular ring; T1: temporal sector of the inner macular ring; S1: superior sector of the inner macular ring; N2: nasal sector of the outer macular ring. I2: inferior sector of the outer macular ring; T2: temporal sector of the outer macular ring; S2: superior sector of the outer macular ring; ST: supero-temporal; SN: supero-nasal; N: nasal; IN: infero-nasal; IT: infero-temporal; T: temporal | | | | | | | | | | | | | | | | | | | | |
|  | | | | | | | | | | | | | | | | | | | | |

**Supplementary table 8. Significant Pearson correlations between RPE and brain structures.**

|  |  | **Retinal layer** | **RPE** | | | | | | | | | | | | | | | | | |
| --- | --- | --- | --- | --- | --- | --- | --- | --- | --- | --- | --- | --- | --- | --- | --- | --- | --- | --- | --- | --- |
|  | **Retinal sector** | **C0** | | **N1** | | **S1** | | **T1** | | **I1** | | **N2** | | **S2** | | **T2** | | **I2** | |  |
|  | **Brain Structure** | **Study group** | **FH– ApoE ɛ4–** | **FH+ ApoE ɛ4+** | **FH– ApoE ɛ4–** | **FH+ ApoE ɛ4+** | **FH– ApoE ɛ4–** | **FH+ ApoE ɛ4+** | **FH–ApoE ɛ4–** | **FH+ ApoE ɛ4+** | **FH– ApoE ɛ4–** | **FH+ ApoE ɛ4+** | **FH-–ApoE ɛ4–** | **FH+ ApoE ɛ4+** | **FH–ApoE ɛ4–** | **FH+ ApoE ɛ4+** | **FH–ApoE ɛ4–** | **FH+ ApoE ɛ4+** | **FH–ApoE ɛ4–** | **FH+ ApoE ɛ4+** |
| **Thickness** | Lingual right | |  |  |  |  |  |  |  |  |  |  |  |  |  |  |  |  |  |  |
|  | Lingual left | |  | -0.492 |  | -0.417 |  | -0.532 |  | -0.453 |  | -0.423 |  |  |  |  |  |  |  |  |
|  | Rostral anterior cingulate right | | -0.432 |  |  |  |  |  |  |  | -0.431 |  |  |  |  |  |  |  |  |  |
|  | Rostral anterior cingulate left | |  |  |  |  |  |  |  |  |  |  |  |  |  |  |  |  |  |  |
|  | Caudal anterior cingulate right | |  |  |  |  |  |  |  |  |  |  |  |  |  |  |  |  |  |  |
|  | Caudal anterior cingulate left | |  |  |  |  |  |  |  |  |  |  |  |  |  |  |  |  |  |  |
|  | Entorhinal left | |  |  |  |  |  |  |  | -0.420 |  | -0.389 |  |  |  |  |  | -0.390 |  |  |
|  | Fusiform right | |  | -0.416 |  | -0.624 |  | -0.532 |  | -0.482 |  | -0.452 |  | -0.554 |  | -0.706 |  |  |  | -0.519 |
|  | Fusiform left | |  | -0.411 |  |  |  |  |  |  |  |  |  | -0.395 |  | -0.539 |  |  |  |  |
|  | Cortical thickness right | |  | -0.469 |  |  |  |  |  |  |  |  |  |  |  |  |  |  |  |  |
|  | Cortical thickness left | |  | -0.452 |  |  |  |  |  |  |  |  |  |  |  |  |  |  |  |  |
| **Volume** | Amygdala left | |  |  |  |  |  | -0.400 |  |  |  |  |  |  |  |  |  |  |  |  |
|  | Intracranial | |  |  |  |  |  |  |  |  |  |  |  | -0.405 |  |  |  |  |  |  |
| FH-: participants without a family history of AD; FH+: participants with a family history of AD; ApoE: Apolipoprotein E; right: right hemisphere and left: left hemisphere. C0: central macular sector; N1: nasal sector if the inner macular ring; I1: inferior sector of the inner macular ring; T1: temporal sector of the inner macular ring; S1: superior sector of the inner macular ring; N2: nasal sector of the outer macular ring. I2: inferior sector of the outer macular ring; T2: temporal sector of the outer macular ring; S2: superior sector of the outer macular ring; ST: supero-temporal; SN: supero-nasal; N: nasal; IN: infero-nasal; IT: infero-temporal; T: temporal | | | | | | | | | | | | | | | | | | | | |
