# Supplementary figures and images for: The relationship between retinal layers and brain areas in asymptomatic first-degree relatives of sporadic forms of Alzheimer’s disease: an exploratory analysis
Source: Alzheimers Res Ther. 2022 Jun 4;14:79. doi: 10.1186/s13195-022-01008-5 (PMC9166601; doi:10.1186/s13195-022-01008-5)

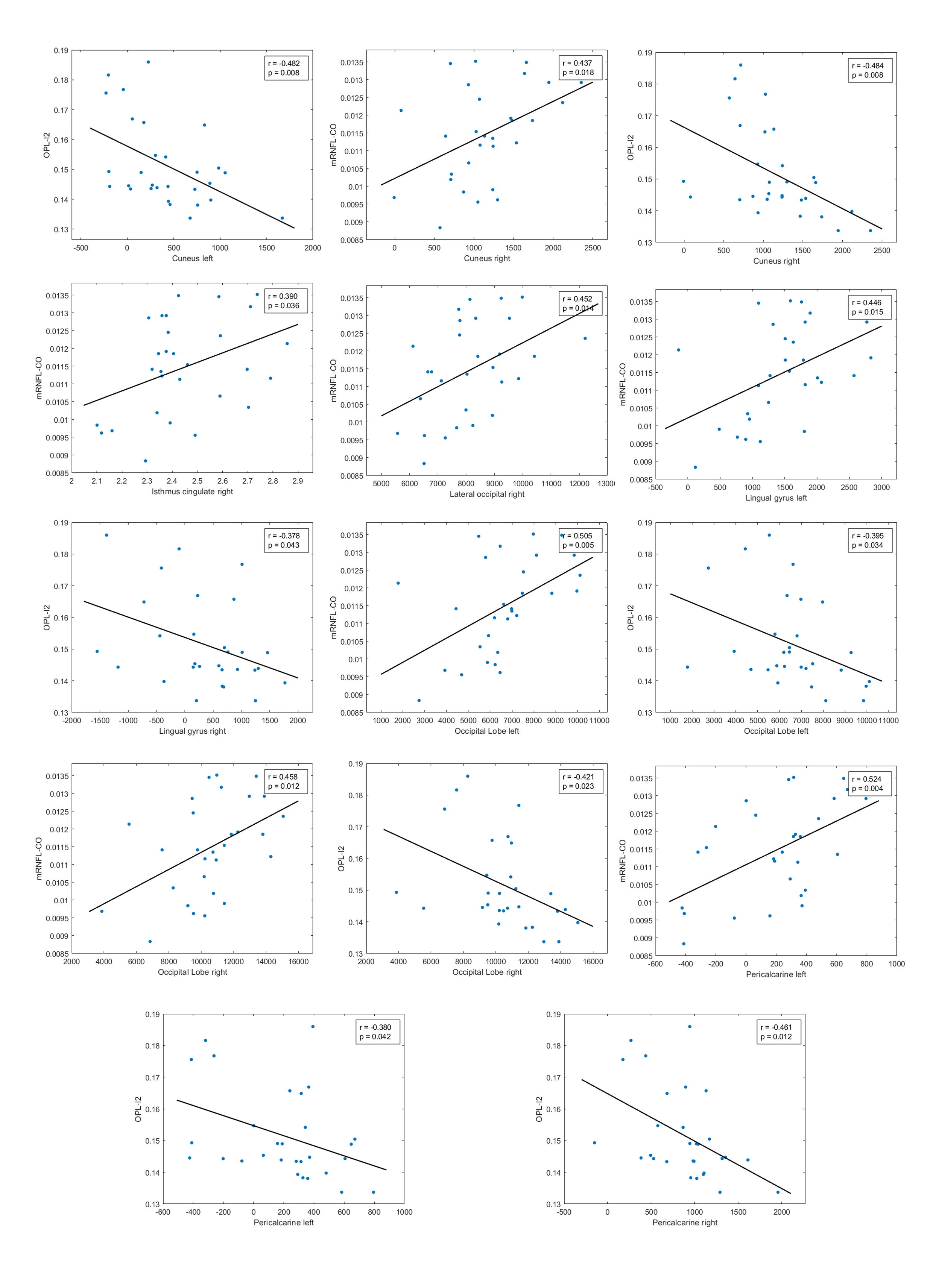

Supplement: Supplementary file 2 — Additional file 2: Figure S1. Scatter plots of statistically significant correlations between retinal sector volumes and volumes and thickness of brain structures in participants with high genetic risk of developing AD. [file 13195_2022_1008_MOESM2_ESM.tif]

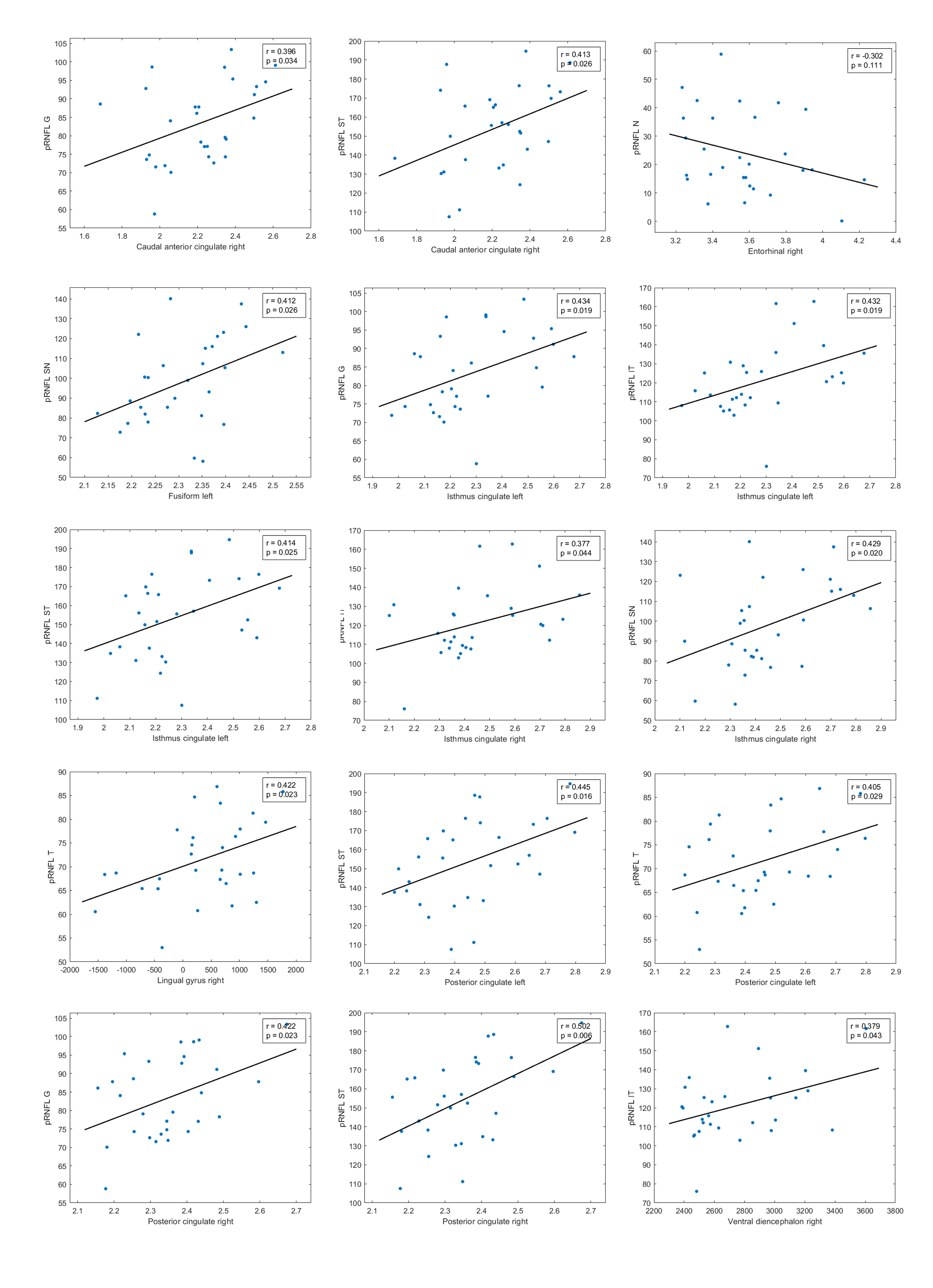

Supplement: Supplementary file 3 — Additional file 3: Figure S2. Scatter plots of statistically significant correlations between peripapillary retinal nerve fiber layer thickness and volumes and thickness of brain structures in participants with high genetic risk of developing AD. [file 13195_2022_1008_MOESM3_ESM.tif]

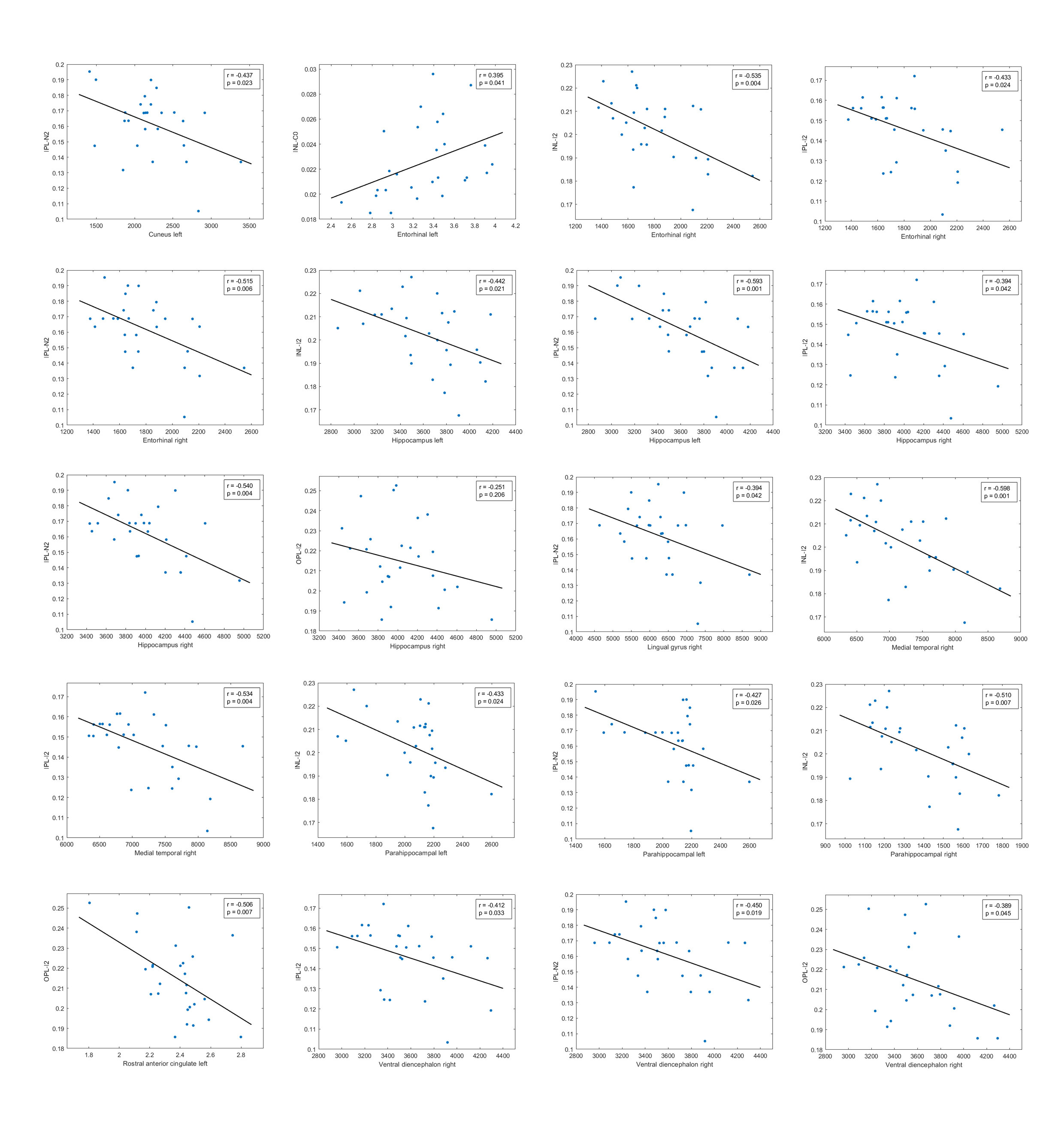

Supplement: Supplementary file 4 — Additional file 4: Figure S3. Scatter plots of statistically significant correlations between retinal sector volumes and volumes and thickness of brain structures in participants without a high genetic risk of developing AD. [file 13195_2022_1008_MOESM4_ESM.tif]

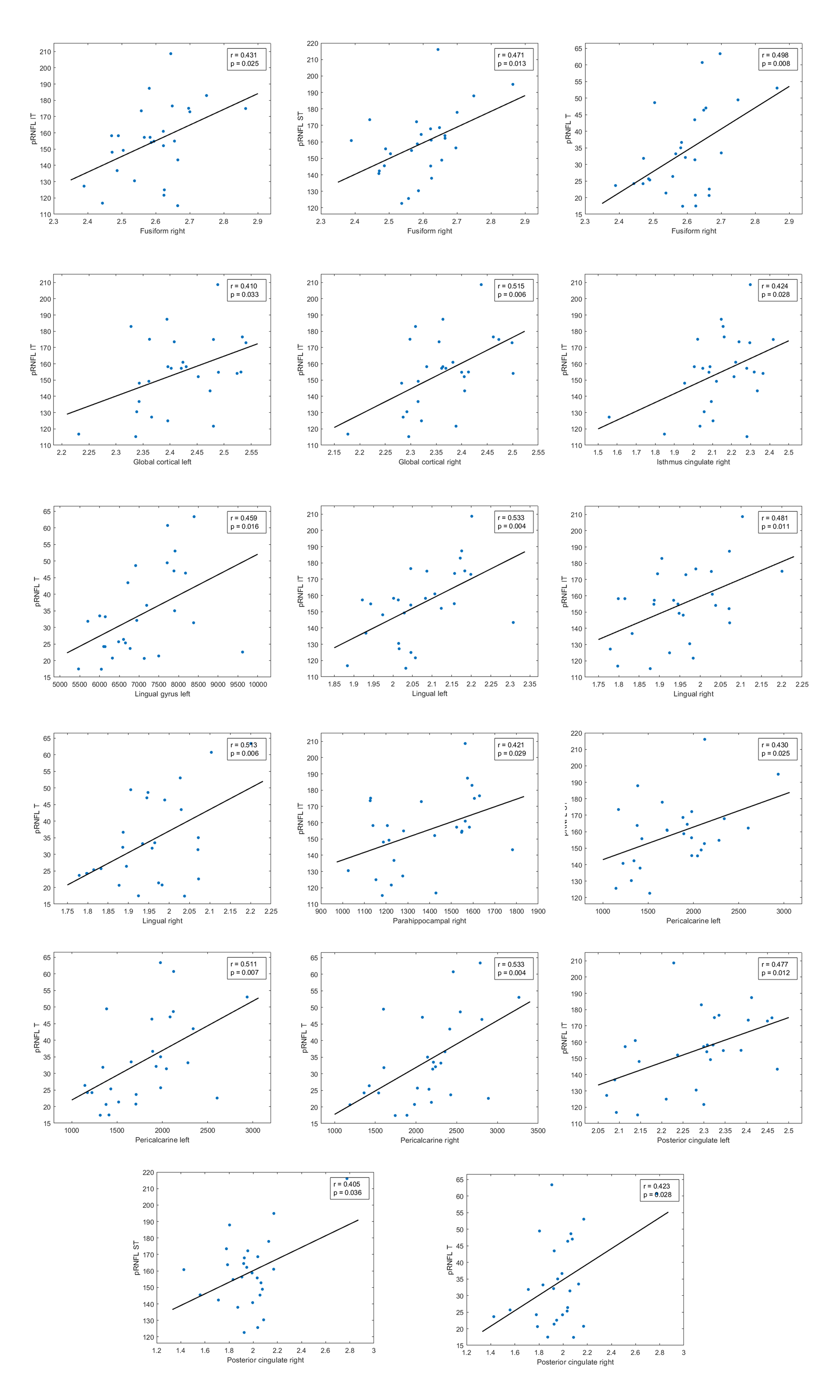

Supplement: Supplementary file 5 — Additional file 5: Figure S4. Scatter plots statistically significant correlations between peripapillary retinal nerve fiber layer thickness and volumes and thickness of brain structures in participants without a high genetic risk of developing AD. [file 13195_2022_1008_MOESM5_ESM.tif]
